# Supplementary material for: Towards fair decentralized benchmarking of healthcare AI algorithms with the Federated Tumor Segmentation (FeTS) challenge
Source: Nat Commun. 2025 Jul 8;16:6274. doi: 10.1038/s41467-025-60466-1 (PMC12238412; doi:10.1038/s41467-025-60466-1)
Supplement: Supplementary file 1 — Supplementary Information [file 41467_2025_60466_MOESM1_ESM.pdf]

Towards Fair Decentralized Benchmarking of Healthcare AI Algorithms with the Federated Tumor Segmentation (FeTS) Challenge

Supplementary Note 1 Additional Dataset Details

To supplement the training data description from the materials and methods, we provide the exact sizes of the training set partitions in Supplementary Fig. 1.

To illustrate the diversity of the task 2 test set, the institution-level meta-data obtained from the collaborators is visualized in Supplementary Fig. 2. Acquisition details were only provided by 20 of 32 sites. In total, 4 different vendors (Siemens, GE, Philips, Hitachi) were used and some institutions acquired with more than one scanner vendor/model. The rough count of unique model names (only considering differences in the first model name part, e.g. Siemens Aera) are: Siemens (12), GE (4), Philips (3), Hitachi (1). Further data heterogeneity is caused by the different acquisition planes (axial/sagittal), field strengths (1.5T/3T) and MRI coils in use. Information about the ethnicity is not available, but geographical diversity could be used as a proxy. It can be seen in fig. 1 of the main manuscript and the exact counts of institutions per continent are: North America (13), Europe (9), South America (4), Asia (3), Africa (1), Australia (1).

The full list of data contributors to the test set for Task 2 in the FeTS challenge 2022, in descending order of sample size, is: Heidelberg University Hospital, Germany; Symbiosis International University, Pune, India / National Institute of Mental Health and Neurosciences, Bengaluru, India; University of Pennsylvania, USA; University of California San Francisco, USA Technical University of Munich, Germany / Klinikum rechts der Isar, Munich, Germany; Leeds Teaching Hospitals Trust; Catalan Institute of Oncology, Spain / Consorci MAR Parc de Salut de Barcelona, Spain / Institute of Diagnostic Imaging - Research Unit, Spain; American College of Radiology, USA; University Hospital of Bern, Switzerland; Thomas Jefferson University, USA; Queen's University, Kingston; Masaryk University / University Hospital Brno, Czech Republic; University of Texas Southwestern Medical Center, USA; Flinders University / South Australia Medical Imaging, Australia; University of Campinas, Brazil; University of Colorado, USA; University of Linz, Austria; The University of Texas MD Anderson Cancer Center, USA; University of Michigan, USA; Yonsei University College of Medicine, Seoul, South Korea; University Sherbrooke, Canada; Luxembourg Institute of Health, Luxembourg; Georgetown University / MedStar Georgetown University

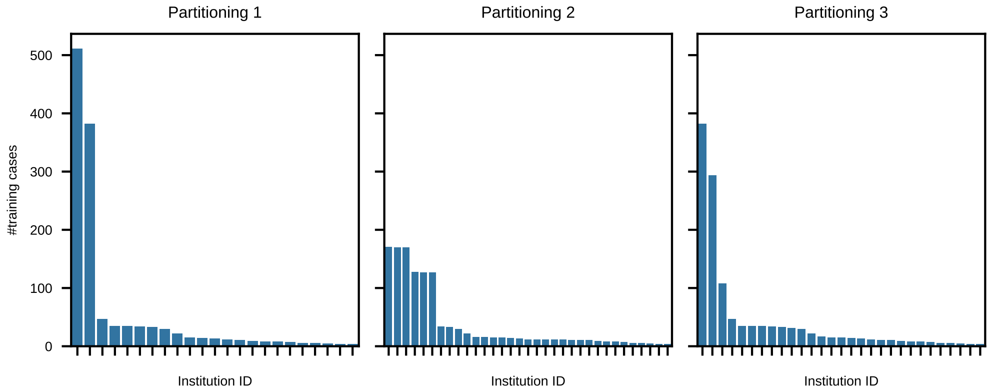

Supplementary Fig. 1: Official partitionings of the training set. Partitioning 1 uses geographical information and 75% of all training cases originate from three locations. Partitioning 2 additionally stratifies the two largest institutions from partitioning 1 by tumor size. Partitioning 3 is a refinement of partitioning 1, which splits the largest institution into 7 based on additional meta-data. Source data are provided as a Source Data file.

Hospital, USA; Tata Memorial Hospital, Mumbai, India; Clínica Imbanaco Quirón Salud / Universidad del Valle, Cali, Colombia; University Hospital Zurich / University of Zurich, Switzerland; Baylor College of Medicine, USA; Universidad de Concepción, Chile; University College Hospital Ibadan, Oyo, Nigeria; Washington University in St. Louis, USA; Escuela Superior Politecnica del Litoral, Ecuador; University of Alabama in Birmingham, USA.

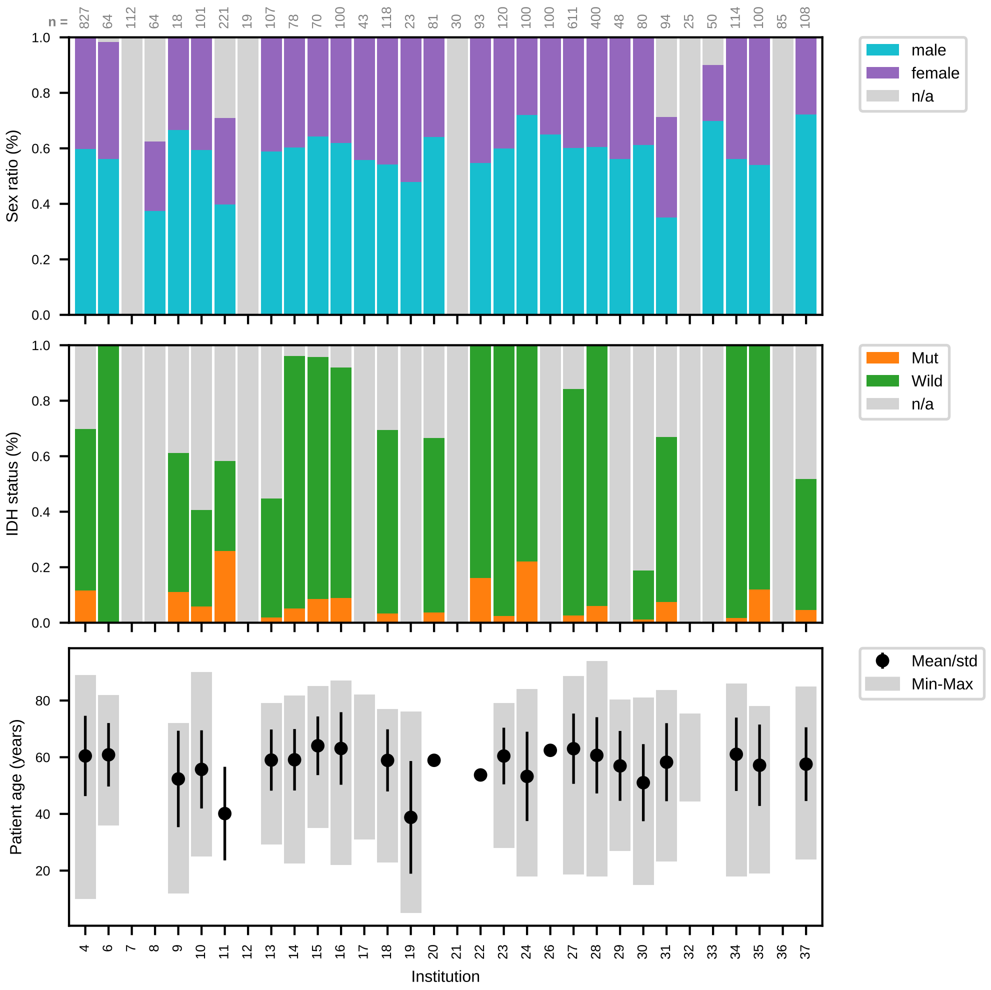

Supplementary Fig. 2: Summary of the meta-data collected from 32 institutions that contributed data to the test data for Task 2. Missing values can occur if institutions did not have the corresponding information for all cases or if they did not report it. For the age distribution, some institutions only provided mean, std or min-max range. The numbers n above each barplot indicate the sample size used to estimate the sex, IDH, and age statistics. These were determined based on numbers collected during the predecessor study [1], so the exact n can be different from the test set sizes but provide a good description of the local population. Boxes for the age distribution indicate minimum and maximum values, while dots and errorbars indicate the mean and standard deviation. Source data are provided as a Source Data file.

Supplementary Table 1: Overview of metric values for each Task 1 team, where ↑/↓ means higher/lower values are better. The mean across 570 test cases is shown here, except for the convergence score, which is a single number per team. Color maps are applied per metric column, ranging from dark red (worst) to dark green (best). Note that the ranking score is not computed from the mean metric values shown here but by case-based ranking as described in the materials and methods. The 'Default' and 'Centralized' models are not ranked, as they are baselines and not part of the competition.

	**DSC ↑**			**HD95 ↓**			**Conv.**	**Rank**
**Team**	WT	TC	ET	WT	TC	ET	**score ↑**	**score ↓**
FLSTAR	75.8	75.1	72.8	31.3	29.9	29.1	72.7	2.75
Sanctuary	76.7	76.3	73.5	24.5	32.6	32.5	71.3	3.05
RoFL	77.5	77.8	74.7	28.9	28.8	29.6	70.2	3.35
gauravsingh	72.4	68.6	66.3	25.2	32.7	32.4	71.5	3.67
rigg	76.8	76.9	74.2	24.4	32.5	32.8	30.1	4.65
HTTUAS	72.5	71.7	67.5	35.9	33.8	35.2	69.0	4.69
Flair	50.5	44.4	50.1	28.6	51.3	44.4	41.8	5.85
Default	74.8	76.2	73.3	37.3	34.0	34.8	69.3	-
Centralized	81.8	80.5	77.2	19.1	31.7	31.3	-	-

Supplementary Note 2 Additional Results Task 1

Comparison to Baselines

A summary of all evaluated methods is shown in Supplementary Table 1, in terms of mean segmentation metrics on the test set, convergence score, and final ranking score. Two baselines, centralized and default, are also included: For the centralized baseline, we only tuned the learning rate and its schedule because the participants were restricted in the same way. However, we did not impose limits on the maximum training time, because such limitations would be unrealistic for centralized training. The validation DSC plateaued in the pooled training after roughly 10 epochs, which is similar to the number of FL rounds feasible within the maximum simulated time with full client participation and one epoch per round. The default FL baseline shows how much the teams could improve their performance during the challenge. It uses FedAvg with the default hyperparameters in the template provided to the challenge participations and selects all sites for training in each FL round. The results for the centralized baseline show that the gap between FL and pooled training is not closed for the FeTS challenge setting. Note that the scores here are not comparable to the Task 2 submissions because different test sets are used and also because Task 2 participants had complete freedom in all algorithmic design choices, while Task 1 participants all used the same segmentation model and local training. The default baseline performs worse than the top teams, especially for the Hausdorff and convergence score metrics, highlighting that client selection and weight aggregation methods can improve performance and efficiency. A comparison with the results of the teams with worse ranking,

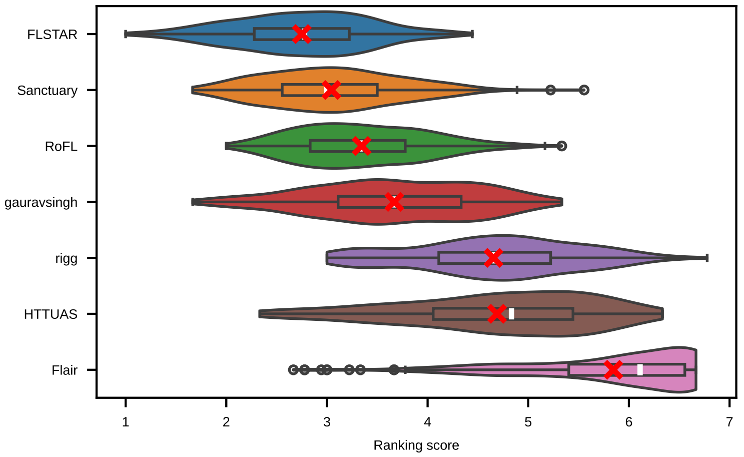

Supplementary Fig. 3: Ranking score of participating teams on the FeTS2022 challenge Task 1 testing cohort. One data point used in this violin plot visualization corresponds to the average ranking for one test case (across DSC, HD95, and convergence score metric). The final ranking is computed as the mean of all ranking scores for each team. Teams are listed in final ranking order. Box plots inside the violin plot indicate median (middle line), 25th, 75th percentile (box), samples within 1.5 times the inter-quartile range (whiskers), and outliers (single points). Source data are provided as a Source Data file.

however, also shows that FedAvg with full participation is still a strong baseline in cross-silo settings, which can outperform more complicated methods in a subset of metrics.

Detailed Metric and Ranking Results

The overall ranking score distribution is shown in Supplementary Fig. 3. This ranking differs from the ranking reported at the MICCAI conference, because the convergence score was initially ranked in reverse order. In the latter, incorrect ranking, the winning team was rigg, followed by RoFL, Sanctuary, HTTUAS, Flair, FLSTAR and gauravsingh. Visualizations of the performance of each team in terms of segmentation metrics can be found in Supplementary Fig. 4.

Details on the Federated Training Runs

The training curves for the best validation DSC over time, which determine the convergence scores, are shown in Supplementary Fig. 5 for all submissions. Additionally, we report the mean and std FL round time for each collaborator in partitioning 3, which was used for the final FL training runs. Supplementary Fig. 6 shows that these are correlated with the number of samples per site.

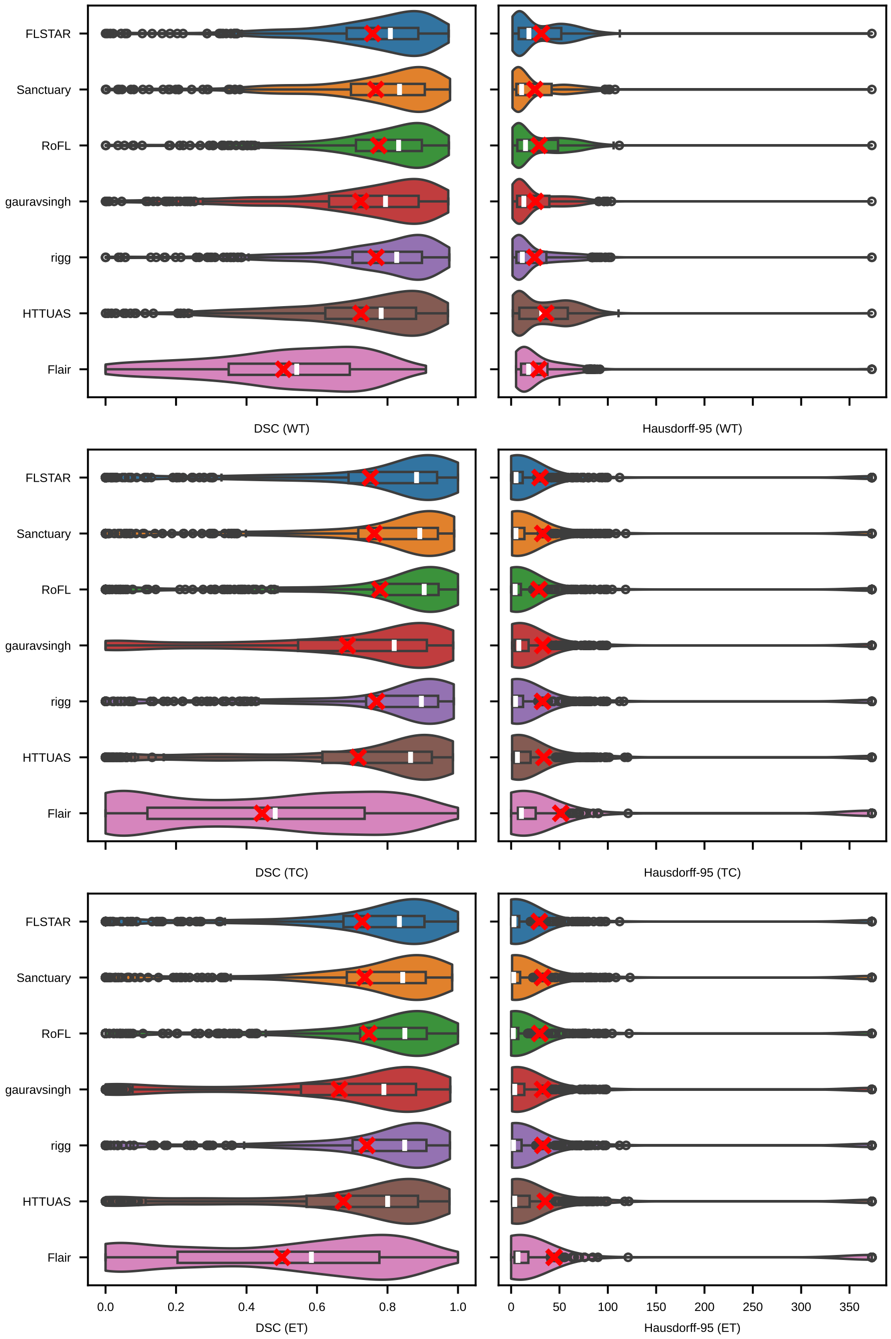

Supplementary Fig. 4: Performance of participating teams on the FeTS2022 challenge testing cohort in terms of DSC coefficient and HD95 distance. Teams are listed on the vertical axis in final ranking order. Inside the violin plots, the mean is shown as a red cross and box plots indicate median (middle line), 25th, 75th percentile (box), samples within 1.5 times the inter-quartile range (whiskers), and outliers (single points).. Source data are provided as a Source Data file.

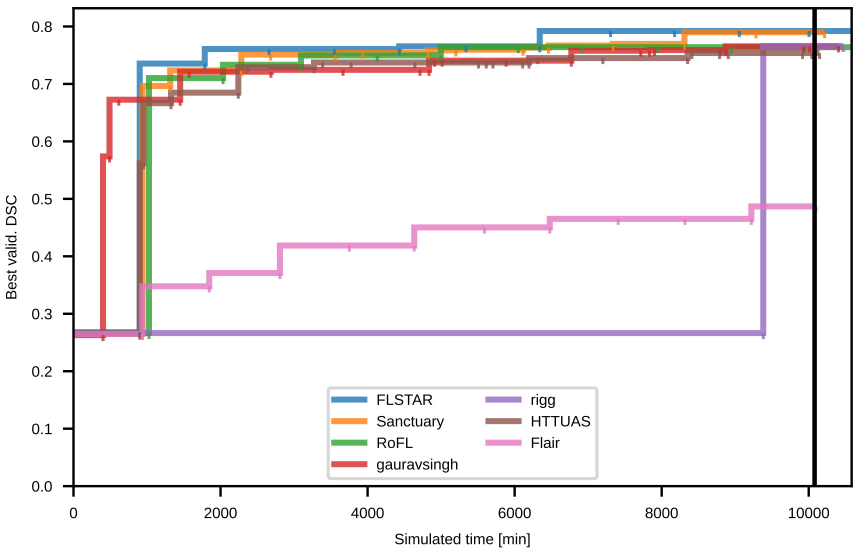

Supplementary Fig. 5: Best validation DSC over simulated FL time for all Task 1 submissions (color-coded). The area under each curve determines the convergence score metric, which measures how quickly the algorithm converges. Tick marks on the curves indicate the end of a communication round and the thick vertical line represents one week of simulated time, after which no checkpoints are saved anymore. Teams are listed in final ranking order. Source data are provided as a Source Data file.

Influence of Hyperparameter Tuning

Apart from collaborator selection and aggregation methods, the FeTS challenge Task 1 further enabled testing of simpler strategies such as tampering with hyperparameters, i.e., variable local epochs in each federated round, decaying learning rates [2–4]. While in some cases these hyperparameters showed some boost, especially the learning rate (schedule), the number of epochs per round was usually set to 1, due to the limited available training time. For Tuladhar et al. [3], extensive tuning did not benefit results on the unseen test sets, which overall seems to indicate that a hyperparameter grid search can be resource-wasting in FL setups.

Supplementary Note 3 Additional Results Task 2

Details on the multi-site evaluation results

The aggregated metric values in fig. 2 of the main article provide an overview of the overall performance. Fig. 3 of the main article zooms into the results for one model, which performed best, but the question remains whether the main findings from this figure hold also for other models. In Supplementary Fig. 7, the distribution of "average case model performance" for each model (e.g., median DSC across all test cases)

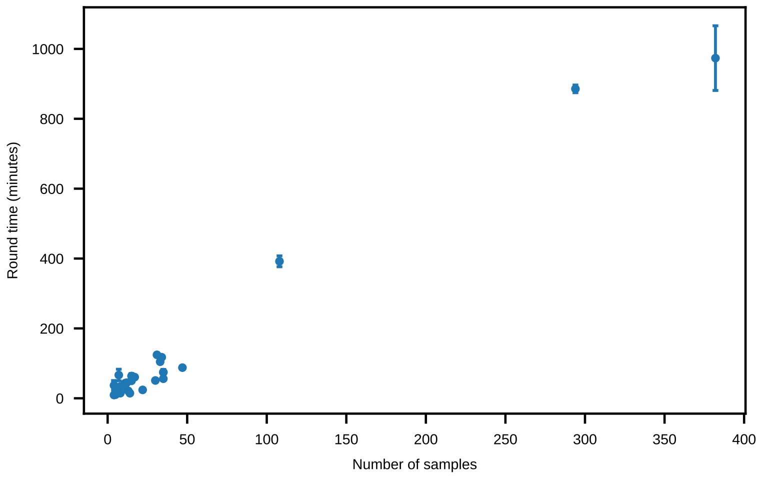

Supplementary Fig. 6: Simulated round time computed for Task 1 compared to the number of samples (patients) for each institution in the training set. Points are placed at the mean round time and errorbars correspond to the standard deviation. Most of the 29 collaborators from partitioning 3 have less than 50 cases and their FL round times are, therefore, considerably shorter than the largest sites. Source data are provided as a Source Data file.

and the "worst case performance" for each model (e.g., 10th percentile of DSC across all test cases) is shown for each institution. This shows that the median performance is very similar between models. For the "worst-case" performance, the differences are bigger, but the overall trend is still comparable for most institutions. Hence, this analysis confirms that failure cases occur for most institutions, independently of whether they were seen during training, and that this trend is consistent between different models.

Focusing on the five official challenge submissions, Supplementary Fig. 8 shows the aggregated metric values for each testing institution and submission, i.e., a filtered version of fig. 2 of the main article. As a more fine-grained analysis, we also provide the distribution of metric values for each submission in Supplementary Fig. 9, limiting the displayed institutions for clarity.

Ranking analysis

After ranking all evaluated models (Supplementary Table 2), we performed a ranking stability analysis on the official submissions through the bootstrapping approach proposed by Wiesenfarth et al. [5]. This analysis was applied to each tumor region (WT, TC, ET) and metric (DSC, Hausdorff) separately. The resulting blob plot is shown in Supplementary Fig. 10. This analysis shows that the ranking between models 10 and 11 is unstable, as well as between models 12 and 8. Between those groups and model 54, the ranking for the DSC metric is stable, but model 54 seems to perform better for

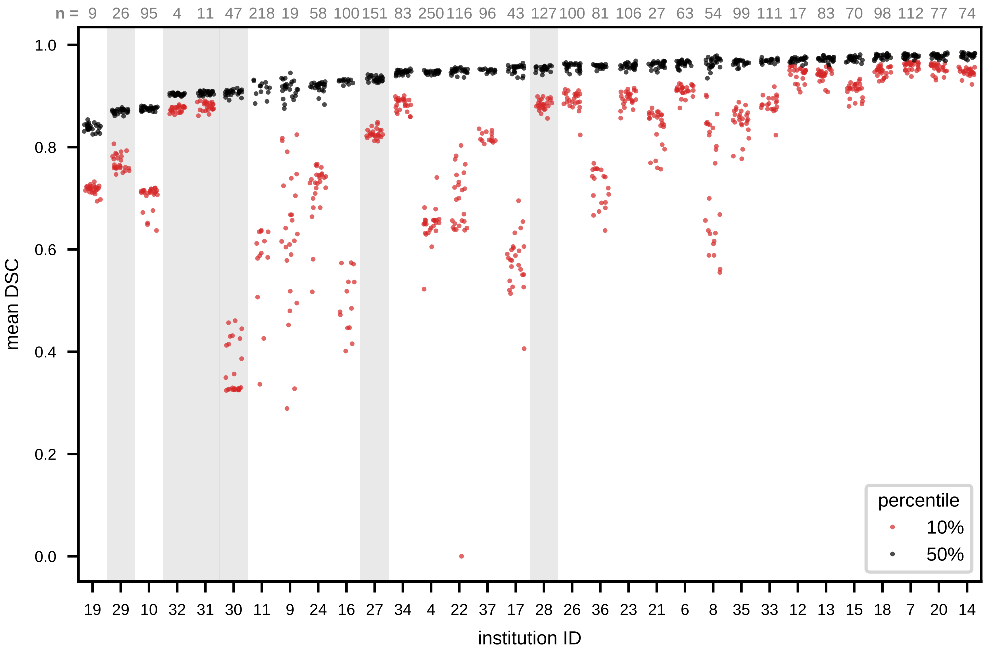

Supplementary Fig. 7: Visualization of per-model performances on test data from each institution. Each dot represents the summarized mean DSC score for one model (10th percentile or median). Gray shading in the background highlights institutions that also contributed (different) cases to the training set. One point corresponds to a single algorithm (only the top-25-ranked are shown) and the n above each column indicates on how many samples the models were evaluated (local testset size). For some institutions, the 10th percentile values are clearly reduced, which highlights a lack of robustness across different models. Source data are provided as a Source Data file.

the Hausdorff metric on average. Together with the well-known fact that the inclusion of additional models in a rank-then-average approach can affect the relative rankings of other models [6], the stability analysis explains the switch of ranks between model 10 and 11 observed in the extended ranking (Supplementary Table 2).

Comparison to BraTS 2021

Here we take a closer look at the test set diversity and compare the performance of submissions on the FeTS2022 and BraTS2021 test sets. Supplementary Fig. 11 shows the number of cases in the FeTS2022 and BraTS2021 test sets split by geographical region. The collaborative, multi-site evaluation in FeTS2022 allowed to increase the test set size and (geographical) diversity significantly, adding new continents (Africa, Australia and South America) to the test set and drastically increasing the total number of samples.

Supplementary Figures 12 and 13 complement fig. 3 of the main article and show the mean segmentation performance across models for each dataset, highlighting which

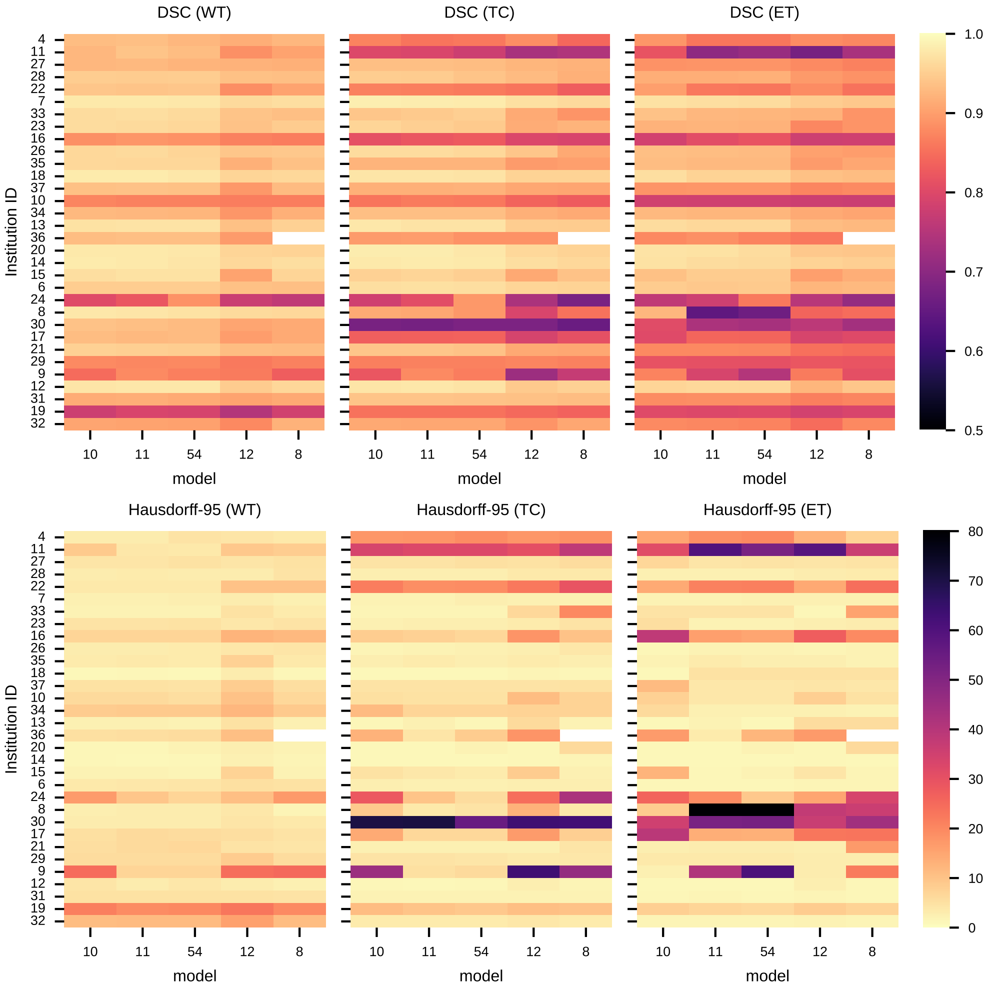

Supplementary Fig. 8: Visualization of aggregated metric values for each testing institution and official FeTS submission. Metrics are aggregated per dataset with mean across the individual cases. The color bar corresponds to the metric printed above each heatmap. For the Hausdorff distance, values are clipped to 80. Source data are provided as a Source Data file.

datasets are part of the BraTS2021 test set and exclusively part of the FeTS2022 test set, respectively. As for the TC region, while there are differences between datasets in general, there is no clear performance gap between the origin of the test set.

Effect of small tumor regions

A known cause of bad segmentation metrics in the BraTS challenge is the presence of empty or very small tumor regions in the reference segmentation. If the reference

Supplementary Table 2: Challenge ranking for Task 2. The MICCAI ranking is computed using only the five official submissions (*model 54 ranked last due to a bug in their official submission, which was fixed later). We also include the extended ranking computed with all available models on the complete FeTS testing data and the ranking difference for each model between the seen-institution subset and unseen-institution subset (cf. fig. 3 of the main article), where a positive/negative value means that an algorithm ranks better/worse on unseen than on seen institutions. Overall the ranking did not change much between these subsets and the models not participating in MICCAI maintained their state of the art.

Model ID	MICCAI rank	Extended rank	Delta BraTS21
10	1	9	−4
11	2	7	2
12	3	28	−2
8	4	27	−2
54	5	8	4
15	–	1	0
35	–	2	0
37	–	3	0
38	–	4	−1
16	–	5	5

segmentation for a tumor region is empty and the prediction is not, the metrics are set to the worst possible value, i.e. 0 for DSC and 373.15 for Hausdorff distance (which is the image diagonal in mm). Even if the reference segmentation is not empty, but very small (e.g. 1 voxel), the DSC score has high variance if the prediction changes only by a few voxels [7]. To investigate the effect of small or empty tumor regions on the evaluation, we performed an analysis where we excluded all cases where the reference segmentation for a tumor region was empty or smaller than 100 voxels (Supplementary Fig. 15). We observe only moderate changes in the metric values, indicating that the effect of small tumor regions is not the main cause of the differences between datasets. Apparently, there are other error sources that lead to low metric values.

Annotation quality control analysis

In the main article results, we described the annotation quality control process that was performed for the FeTS2022 test set, which aimed to select those multicentric annotations for evaluation that are consistent with the shared annotation protocol. As a possible basic improvement on the status quo, we investigated whether the annotation quality control could profit from guidance by a reference algorithm. To this end, we simulated a quality control process where the samples are inspected in the order of the DSC score of a reference algorithm (we used the minimum DSC across the WT, TC, and ET regions for each sample). The results in Supplementary Fig. 17 show a clear improvement compared to random sampling for review.

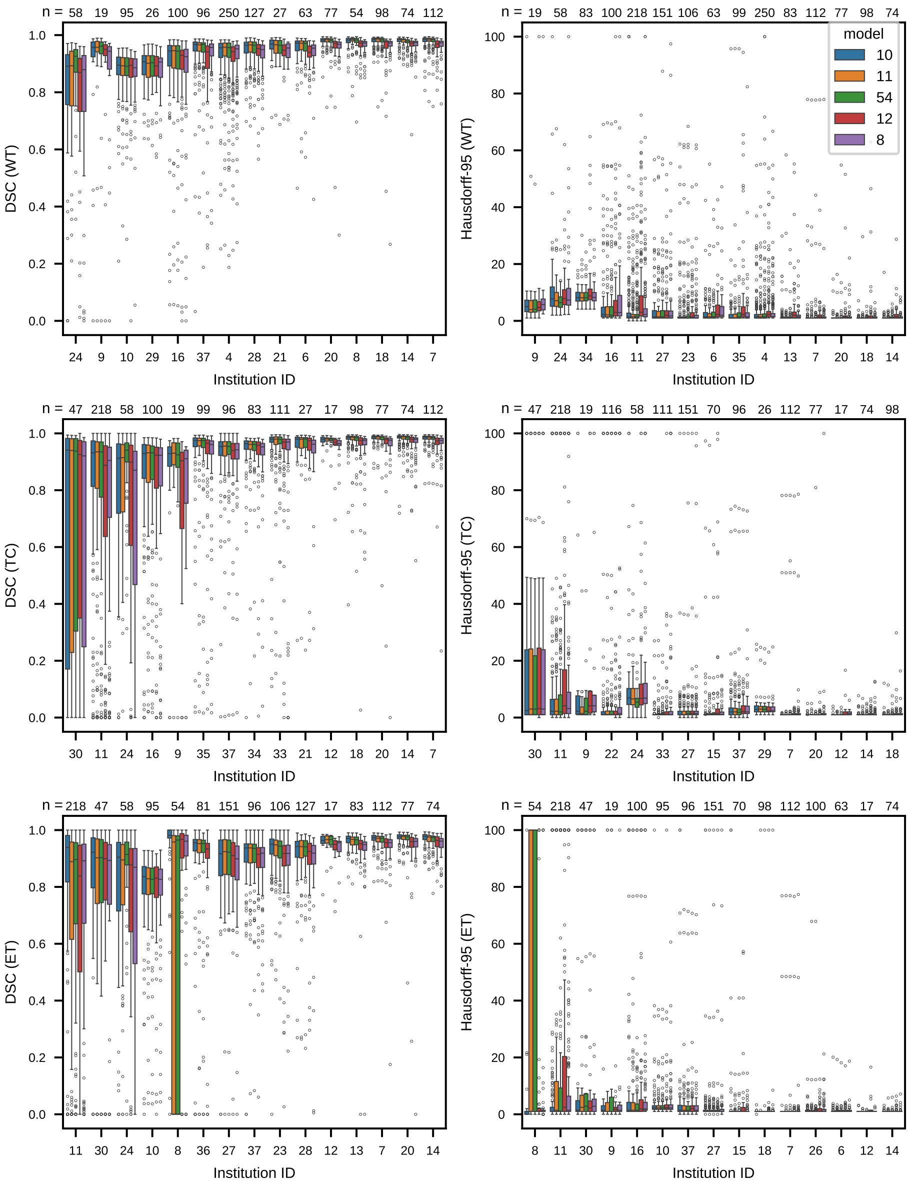

Supplementary Fig. 9: Visualization of all DSC and Hausdorff metrics (clipped to [0, 100]) for the five official FeTS submissions. Box plots indicate median (middle line), 25th, 75th percentile (box), samples within 1.5 times the inter-quartile range (whiskers), and outliers (single points). Only a subset of sites is shown here for clarity, selected by picking (for each region) the sites with top-5, bottom-5, and median-5 performance. Usually, the performance of most models is similar within each dataset and varies much more between different sites. While the median metric values are still high, the box plots for the worst sites (on the left) also show considerable tails in the low-performance region, especially for the DSC metric. Source data are provided as a Source Data file.

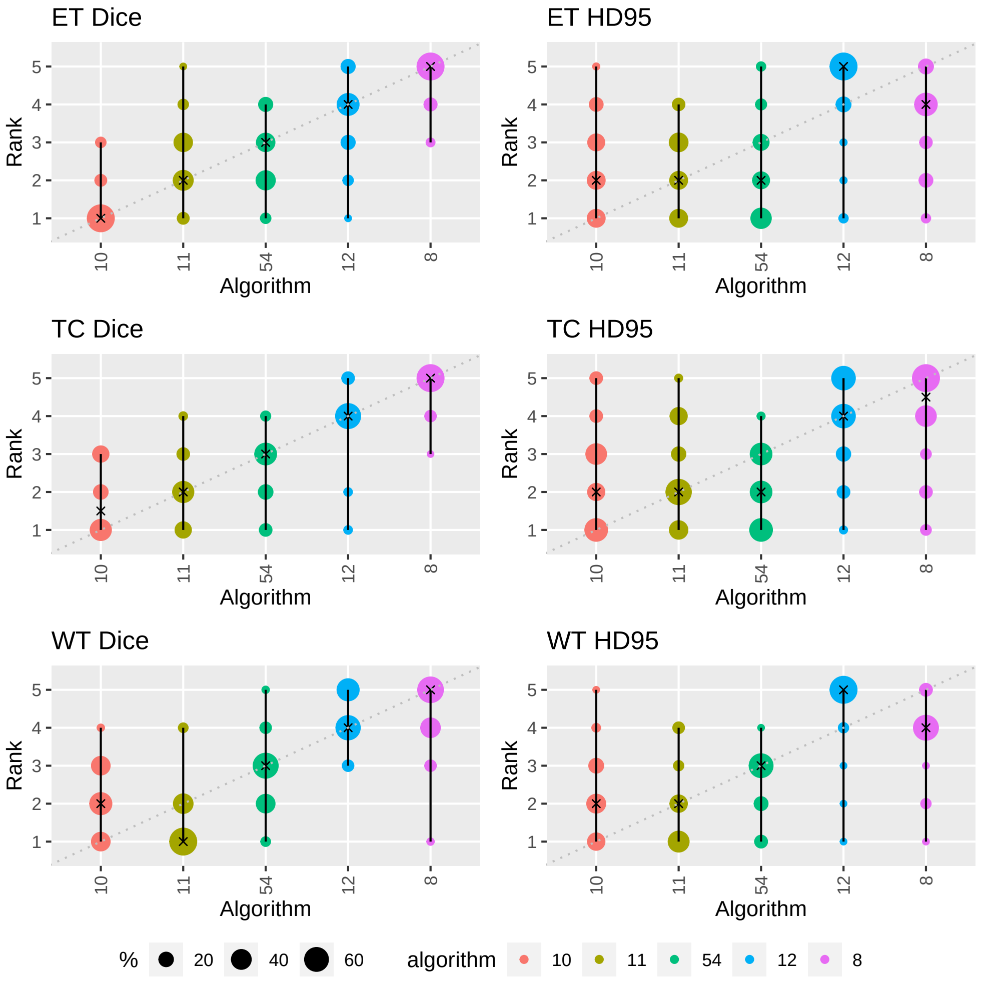

Supplementary Fig. 10: Ranking stability visualization based on 1000 bootstrap samples per institution for each tumor region and metric, computed for the five official challenge submissions. The blob plot shows that the most stable sub-rankings are ET DSC and TC DSC. In the other sub-rankings, algorithms 10 and 11 are close, as well as 12 and 8. Source data are provided as a Source Data file.

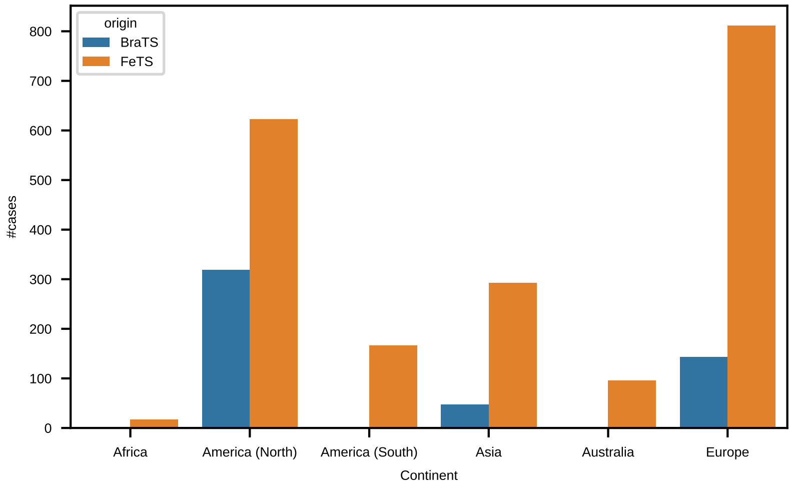

Supplementary Fig. 11: Number of test cases split by geographical region and data source (origin). The multi-site evaluation in FeTS2022 added test cases from the FeTS consortium to the ones already collected for BraTS, which increased the test set size and (geographical) diversity significantly. Source data are provided as a Source Data file.

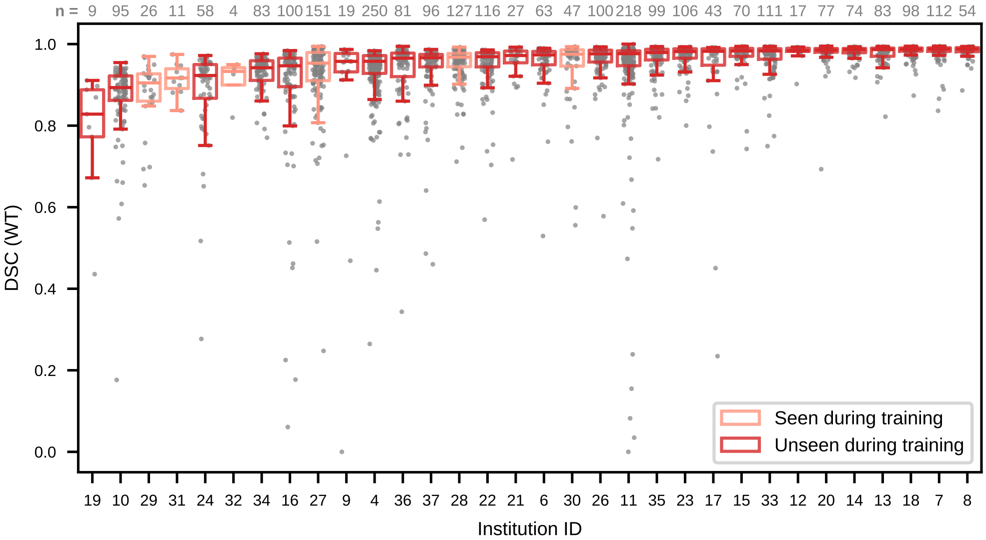

Supplementary Fig. 12: Similar to fig. 3 of the main article, but for the WT region: Performance of the top-ranked algorithm (ID 15) for each institution of the test set. Box plots indicate median (middle line), 25th, 75th percentile (box), and samples within 1.5 times the inter-quartile range (whiskers). Some institutions contributed distinct patients to both the training and testing dataset ("seen during training"), while others were unseen before testing. Each grey dot represents the DSC score of the WT region for a single test case (results for individual tumor regions in Supplementary Figures 12 to 14). Institutions with reduced performance or outlier cases exist both within the BraTS subset and the FeTS subset. Notably, even institutions seen during training may in some cases exhibit large performance drops on the test set. Source data are provided as a Source Data file.

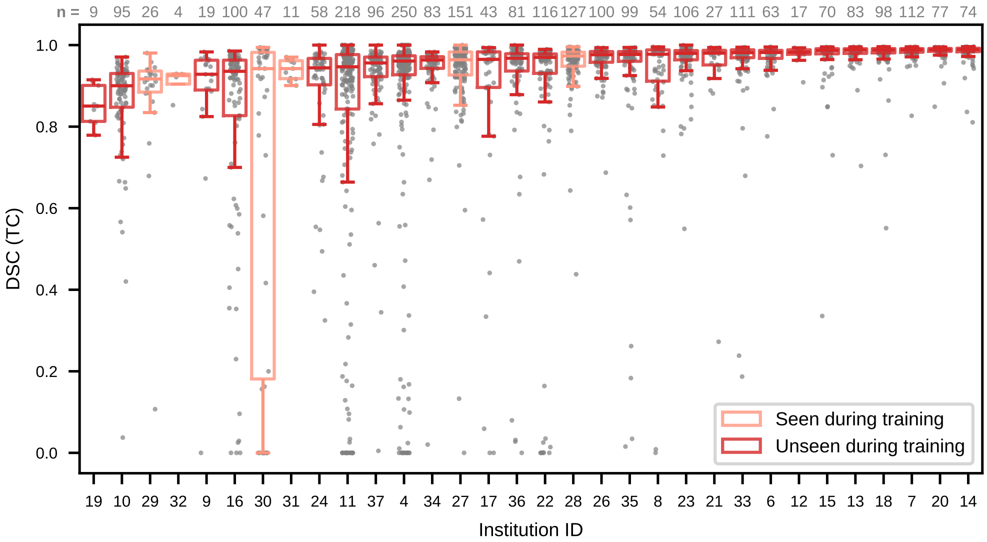

Supplementary Fig. 13: Similar to fig. 3 of the main article, but for the TC region: Performance of the top-ranked algorithm (ID 15) for each institution of the test set. Box plots indicate median (middle line), 25th, 75th percentile (box), and samples within 1.5 times the inter-quartile range (whiskers). Some institutions contributed distinct patients to both the training and testing dataset ("seen during training"), while others were unseen before testing. Each grey dot represents the DSC score of the TC region for a single test case (results for individual tumor regions in Supplementary Figures 12 to 14). Institutions with reduced performance or outlier cases exist both within the BraTS subset and the FeTS subset. Notably, even institutions seen during training may in some cases exhibit large performance drops on the test set. Source data are provided as a Source Data file.

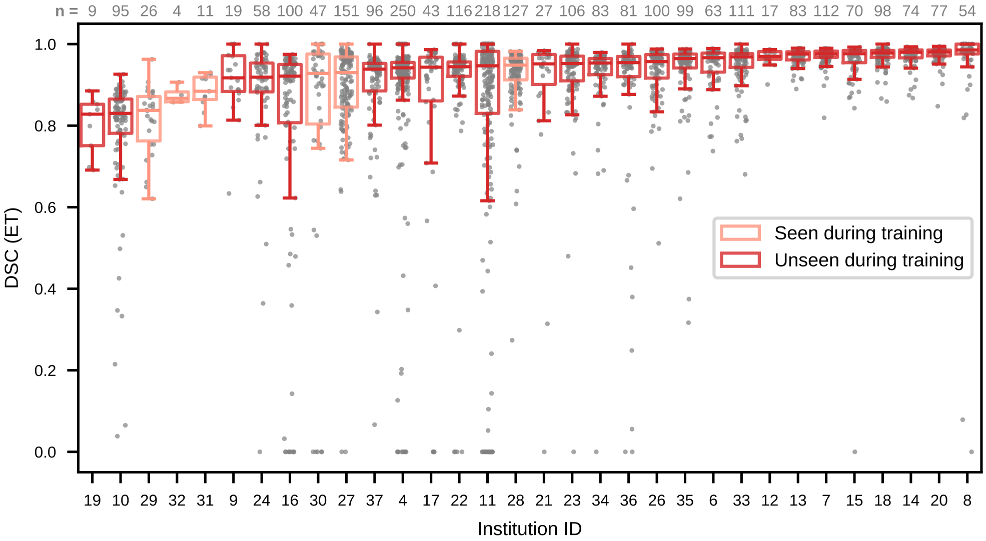

Supplementary Fig. 14: Similar to fig. 3 of the main article, but for the ET region: Performance of the top-ranked algorithm (ID 15) for each institution of the test set. Box plots indicate median (middle line), 25th, 75th percentile (box), and samples within 1.5 times the inter-quartile range (whiskers). Some institutions contributed distinct patients to both the training and testing dataset ("seen during training"), while others were unseen before testing. Each grey dot represents the DSC score of the ET region for a single test case (results for individual tumor regions in Supplementary Figures 12 to 14). Institutions with reduced performance or outlier cases exist both within the BraTS subset and the FeTS subset. Notably, even institutions seen during training may in some cases exhibit large performance drops on the test set. Source data are provided as a Source Data file.

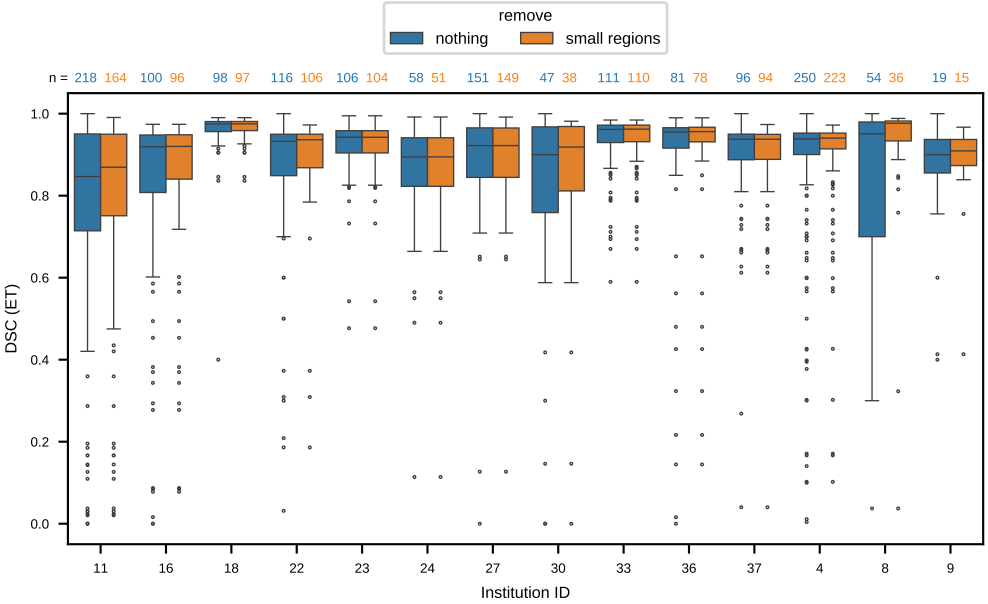

Supplementary Fig. 15: Effect of empty/small tumor regions in reference segmentation. Box plots indicate median (middle line), 25th, 75th percentile (box), samples within 1.5 times the inter-quartile range (whiskers), and outliers (single points). As enhancing tumor (ET) is usually the smallest region, the diagram shows the DSC values, averaged across the top-10 models per case, before and after cases with ET region volume $< 100\,\mathrm{mm}^3$ are removed from the evaluation. Only institutions that contain such cases are shown. The results become less extreme and medians improve slightly, but the differences between institutions remain and there are still many outlier cases for all datasets. Source data are provided as a Source Data file.

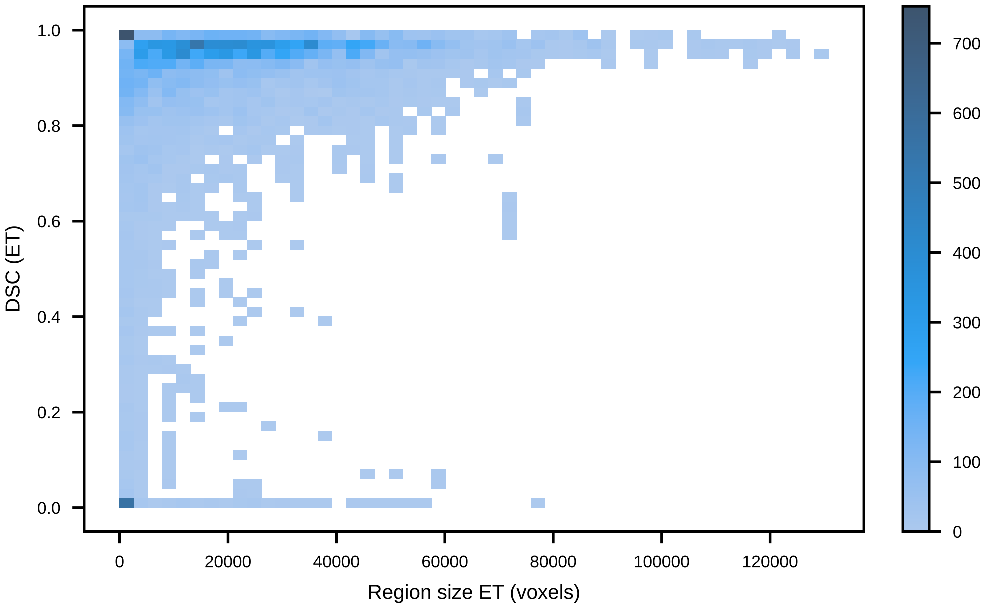

Supplementary Fig. 16: 2D-Histogram of GT region size vs. metric for all datasets and top-10 models. The ET region is shown as it is the smallest tumor region. For small structures, models usually achieve lower DSC scores. For empty (size = 0) regions, models can either get a DSC of 0 or 1, which explains the accumulations at these points in the diagram. However, the cases with small or empty regions still represent a minority of the data. Source data are provided as a Source Data file.

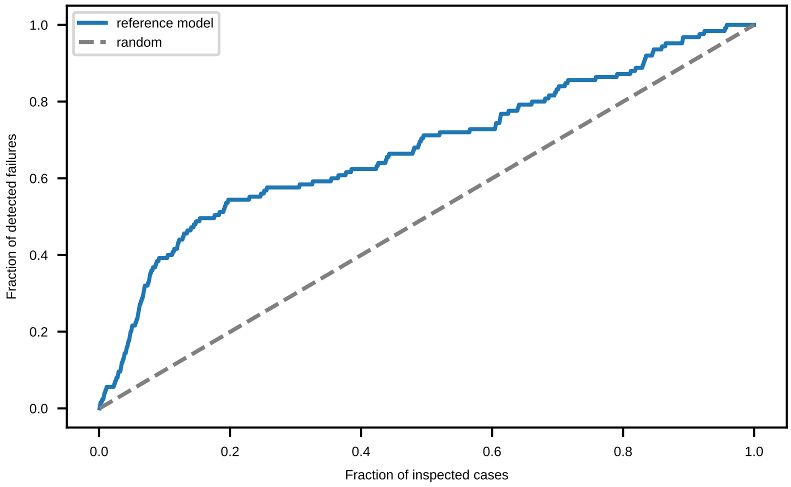

Supplementary Fig. 17: Simulation of quality control screening guided by a reference algorithm. Inspecting samples after ordering them according to the DSC score of a reference algorithm (we used the minimum DSC across the WT, TC, and ET regions for each sample) results in more failures being detected than inspecting them in random order. This approach could be used to speed up the quality control process in other applications where SOTA models are available. Source data are provided as a Source Data file.

Supplementary Table 3: Participation and team details for the FeTS challenge 2022. The model ID is given in the task columns. Only a single affiliation is given for consecutive authors with the same institution.

Team Name	Task 1	Task 2	Members
Flair	Yes	-	Vasilis Siomos (University of London)
FLSTAR	Yes	-	Yuan Wang*, Renuga Kanagavelu, Qingsong Wei, Yechao Yang (Institute of High Performance Computing)
gauravsingh	Yes	-	Gaurav Singh (IIITV)
Graylight Imaging	-	11	Krzysztof Kotowski, Szymon Adamski, Bartosz Machura, Wojciech Malara (Graylight Imaging), Lukasz Zarudzki (Maria Sklodowska-Curie Memorial Cancer Center and Institute of Oncology), Jakub Nalepa* (Silesian University of Technology)
HPCASUSC	-	12	Yaying Shi (University of North Carolina at Charlotte)*, Hongjian Gao, Salman Avestimehr (University of Southern California), Yonghong Yan (University of North Carolina at Charlotte)
HT-TUAS	Yes	-	Muhammad Irfan Khan (Turku University of Applied Sciences)*, Mohammad Ayyaz Azeem (Riphah International University), Esa Alhoniemi, Elina Kontio, Suleiman A. Khan, Mojtaba Jafaritadi (Turku University of Applied Sciences)
NG research	-	9/54	Jianxun Ren*, Wei Zhang, Ning An (NG research), Qingyu Hu (University of Science and Technology of China), Youjia Zhang (Neural Galaxy), Ying Zhou (NG research)
rigg	Yes	-	Leon Mächler (ENS Paris)*; Ivan Ezhov; Suprosanna Shit; Johannes C. Paetzold (TUM)
RoFL	Yes	-	Ambrish Rawat*; Giulio Zizzo (IBM Research); Swanand Kadhe (UC Berkeley); Jonathan P Epperlein; Stefano Braghin (IBM Research)
Sanctuary	Yes	10	Meirui Jiang (The Chinese University of Hong Kong)*; Hongzheng Yang (Beihang University); Xiaofan Zhang ; Shaoting Zhang (Shanghai Artificial Intelligence Laboratory); Qi Dou (The Chinese University of Hong Kong)
vizviva	-	8	Himashi Peiris*; Munawar Hayat ; Zhaolin Chen; Gary Egan; Mehrtash Harandi (Monash University, Australia)

Supplementary Note 4 Details of Participating Teams

The methods description of all participating teams is included in the materials and methods section of the main article. Here, we provide a list of team members for the official submissions in Supplementary Table 3 and a mapping from additional BraTS 2021 algorithms evaluated in Task 2 to the corresponding publications describing their methodology in Supplementary Table 4.

Supplementary Table 4: Mapping from algorithm ID to scientific publication for the subset of BraTS 2021 models evaluated within the FeTS challenge. Submissions are listed in ranking order of the BraTS 2021 challenge and the top 10 are marked bold.

ID	Reference	ID	Reference
**15**	Luu and Park [8]	**35**	Yuan [9]
**14**	Futrega et al. [10]	**37**	Ma and Chen [11]
**16**	Kotowski et al. [12]	**51**	Jia et al. [13]
**38**	Dobko et al. [14]	**33**	Alam et al. [15]
**31**	Nguyen-Truong and Pham [16]	**46**	Fidon et al. [17]
27	Yang et al. [18]	40	Jiang et al. [19]
18	Wu and Lin [20]	19	Zeineldin et al. [21]
42	n/a (team *tigerduck*)	32	Carré et al. [22]
44	Pnev et al. [23]	25	Feng et al. [24]
48	Singh [25]	30	Pawar et al. [26]
13	n/a (team *younet*)	26	Bukhari and Mohy-ud Din [27]
41	Milesi et al. [28]	45	Demoustier et al. [29]
36	Shah et al. [30]	22	Li et al. [31]
52	Akbar et al. [32]	23	Yang et al. [33]
43	Maurya et al. [34]	39	Lin et al. [35]
21	Roth et al. [36]	28	Saueressig et al. [37]
29	Yan et al. [38]	24	n/a (team *Team Two*)
53	Hsu et al. [39]	47	Druzhinina et al. [40]

Supplementary Note 5 Comparison to FeTS Challenge 2021

Challenge Datasets

The imaging modalities, annotation characteristics, and preprocessing are identical to the FeTS 2022 challenge. However, the FeTS 2021 challenge used the BraTS 2020 dataset as a data source, which consisted of 341 training cases and 112 validation cases. For the testing phase, Task 1 used 166 cases from the BraTS 2020 test set, while Task 2 evaluated submissions on 545 cases and 21 institutions from the FeTS [1] federation. Hence, the FeTS challenge 2022 has a significantly larger training and test set (Supplementary Fig. 18).

Organization

Only technical details differed between the FeTS 2021 and 2022 challenges. For Task 1, the federated learning framework was updated and the participant interface was improved. For Task 2, in 2021 a Python script for submission download and evaluation was integrated into a custom version of the FeTS tool [1], which was installed at the participating sites. This was replaced in 2022 by MedPerf [41] for improved functionality and usability.

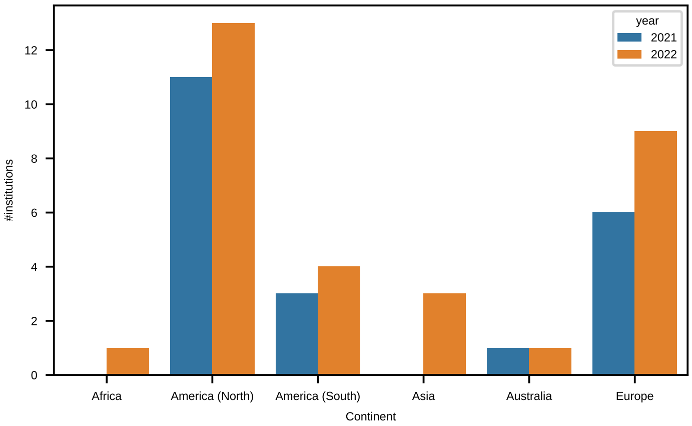

Supplementary Fig. 18: Number of distinct institutions contributing to the FeTS challenge 2021/2022 test sets. Two new continents (South America and Africa) were added to the test set in 2022 and the total number of test cases increased from 545 to 2638 (the median number of cases per institution grew from 17 to 85). Source data are provided as a Source Data file.

Results

Task 1

Raw segmentation metric values for each team are visualized in Supplementary Fig. 19, the convergence scores are depicted in Supplementary Fig. 20, and the ranking scores in Supplementary Fig. 21. Note that the rankings were recomputed for this manuscript with the same weighting (3) for the convergence score, for consistency with the FeTS 2022 challenge, so the ranking is slightly different than the one presented at MICCAI 2022.

Task 2

The mean metric values per dataset and model show clear performance differences between datasets, similar to the results of the FeTS 2022 challenge in fig. 2 of the main article. Datasets C08, C27, and C15 stand out in particular, with DSC scores below 0.7 for all tumor regions. However, low Hausdorff distances for models A1 and A2 indicate that the segmentation boundaries are still close to the reference mask. The small sample size and impossibility of inspecting algorithm prediction masks distributed among the evaluation sites in 2021 prevented us from investigating the reasons for the low performance in these datasets. Regarding ranking, differences between the three submitted algorithms were larger in 2021. This is reflected by the ranking stability analysis in Supplementary Fig. 23, for which pairwise significance testing between algorithms is performed following Wiesenfarth et al. [5] for each combination of institution, tumor region and metric ($= 21 \cdot 3 \cdot 2$) and the resulting significance counts are summed.

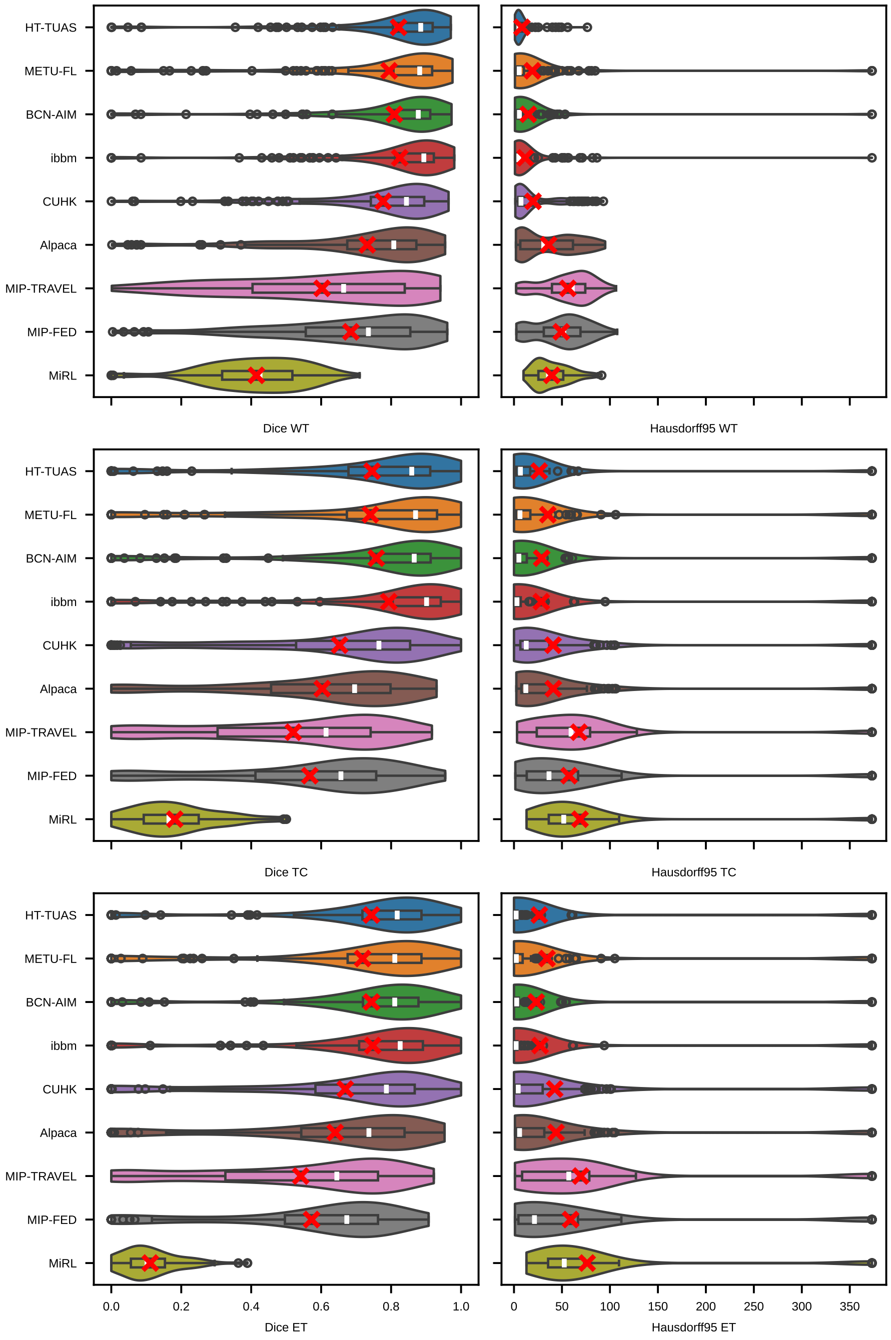

Supplementary Fig. 19: Performance of participating teams on the FeTS2021 challenge testing cohort in terms of DSC coefficient and HD95 distance. Teams are listed in final ranking order. The mean is shown as a red cross and box plots indicate median (middle line), 25th, 75th percentile (box), samples within 1.5 times the inter-quartile range (whiskers), and outliers (single points). Source data are provided as a Source Data file.

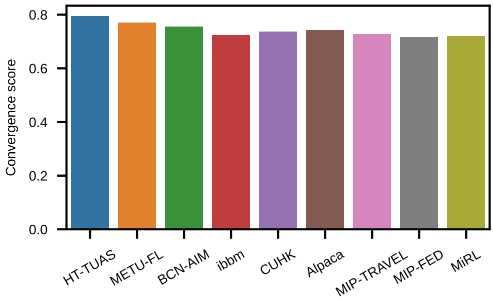

Supplementary Fig. 20: Convergence score of participating teams on the FeTS2021 challenge testing cohort, defined as the area under the validation DSC curve. Teams are listed in final ranking order. Source data are provided as a Source Data file.

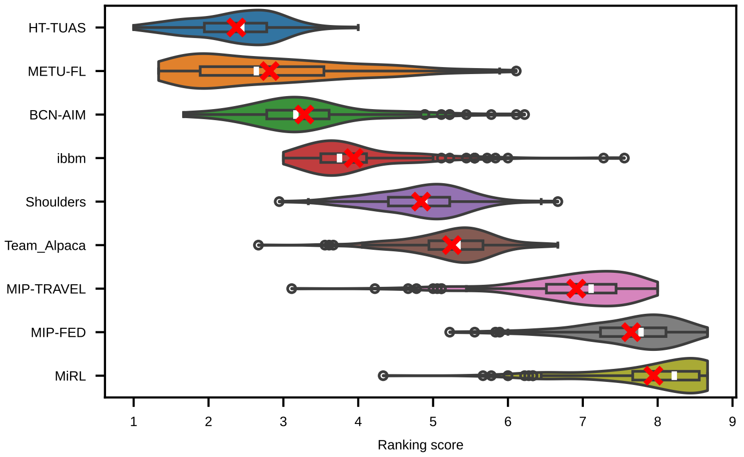

Supplementary Fig. 21: Ranking score distribution across test cases of all participating teams on the FeTS2021 challenge Task 1 testing cohort. One data point used in this violin plot visualization corresponds to the average ranking for one test case (across DSC, HD95, and convergence score metric). The final ranking is computed as the mean of all ranking scores for each team. Teams are listed in final ranking order. The mean is shown as a red cross and box plots indicate median (middle line), 25th, 75th percentile (box), samples within 1.5 times the inter-quartile range (whiskers), and outliers (single points). Source data are provided as a Source Data file.

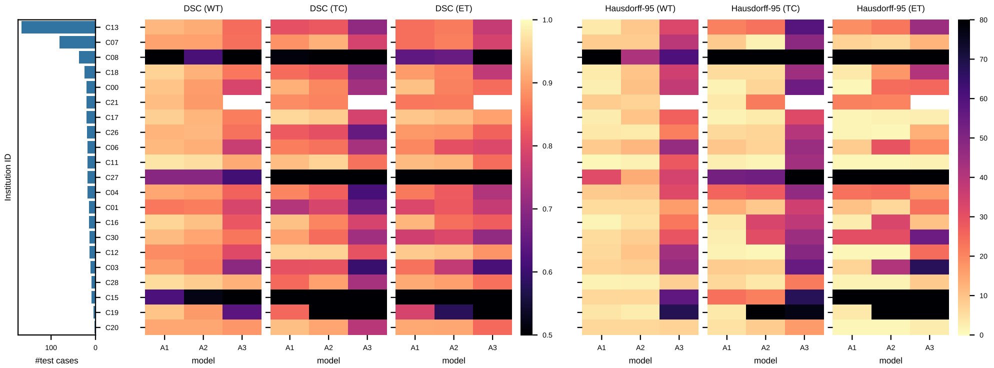

Supplementary Fig. 22: Aggregated (mean) metric values for FetS 2021. White color indicates failed evaluation (technical error of the submission), and black color a metric value that extends beyond the color bar limits (i.e. DSC < 0.5 or HD95 > 80). Source data are provided as a Source Data file.

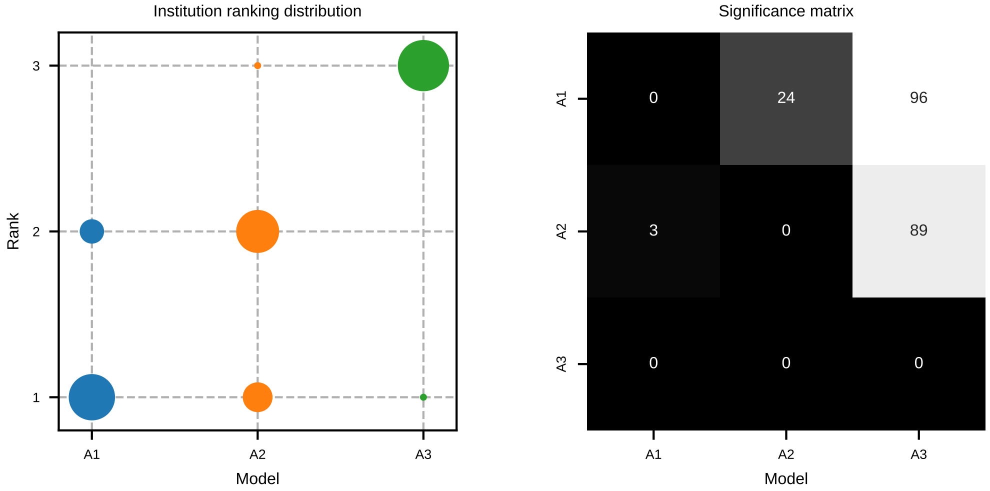

Supplementary Fig. 23: Ranking results of the FeTS challenge Task-2 in 2021. In the Blob plot of per-institution rankings (left), the size of the markers is proportional to the ranking count ($N = 21 \cdot 3 \cdot 2 = 126$ sub-rankings in total per model). A1 performs overall best, followed by A2. A test-based ranking stability analysis (right) confirms the clear ranking, as A1 is superior to A2 in 24 sub-rankings and only 3 vice versa. The one-sided Wilcoxon signed rank test at 5% significance level with adjustment for multiple testing according to Holm was employed (following [5]) for each per-institution ranking. Source data are provided as a Source Data file.

Participating teams

An overview is given in Supplementary Table 5.

Team Alpaca [42]

A new weight-aggregation logic was developed based on the average validation DSC scores of each collaborator. Hyper-parameters used for training the network were selected based on the performance of the previous round of federated training. For the first 5 rounds, the learning rate was set to 1e-3 and epochs per round to 10. After 5 rounds, these hyper-parameters were kept constant if the calculated average DSC score was ≤ 0.5, changed to 1e-4 and 5 if it was > 0.5, and the learning rate was further reduced to 1e-5 if it was > 0.8.

Team BCN-AIM [43]

Center Dropout is a simple method for sampling centers during each federated round. Concretely, it selects a random subset of centers in each round, that correspond to a total percentage of the data defined by the user. The selection process of the centers may be entirely random or guided by elements such as center speed or aleatoric uncertainty. The method achieves increased speed because the slowest centers are not present in every iteration, and increased fairness because the largest centers do not always overwhelm the voting process during aggregation.

Team CUHK [44]

Solution Task 1: Considering that the clients participating in federated optimization are highly heterogeneous in the number of local stochastic gradient descent iterations, we design an effective tensor normalization approach to re-normalize aggregated tensors for stabilizing the federated learning process. Besides, we also devise a client pruning strategy to adaptively filter out the slower clients, which harms the parallel performance of federated learning thus incurring a slower convergence rate.

Solution Task 2: Since the samples from unseen data sources usually present apparent distribution shifts due to the varying image acquisition conditions, a test-time adaptation mechanism is used to dynamically adjust the model parameters at test time. Since the higher entropy of model predictions usually reflects a notable domain shift, the unsupervised objective for model parameter adaptation is minimization of the prediction entropy of test samples.

Team HT-TUAS [45]

The HT-TUAS team presented the Similarity Weighted Aggregation (SimAgg) method for the FeTS2021 competition, focusing on batch-wise collaborator selection and parameter aggregation policy. Collaborators are randomly selected (e.g., 20%) in each round, ensuring heterogeneous participation. A sliding window maintains equal collaborator involvement. After each round, a new random order is generated for improved learning. The chosen aggregation policy addresses diverging weights and parameters

using a weighted aggregate at the server. Collaborators are weighted based on similarity to the non-weighted average, promoting a master model that represents the majority. Close collaborators receive higher similarity scores, while a second factor favors those with larger sample sizes. Normalized aggregated parameters are then sent to the next batch for subsequent rounds.

Team ibbm [46]

Their proposed concept is inspired by a PID controller. This new weighting strategy not only adjusts for the different local training data set sizes but also for the different amounts of local progress that were made during the last federated training round. The weight of each local model is a linear combination of the relative sizes of local training data sets as well as the amounts by which the local costs decreased during the last round, where local updates which only marginally improved the local cost get lower weight.

Team mbi [47]

A deep learning method is proposed for brain tumor segmentation using a two-stage orthogonal encoder-decoder convolutional neural network (CNN). An orthogonal network is an ensemble of three encoder-decoder networks trained on axial, sagittal, and coronal slices. The proposed method is a two-stage network: in stage-I, a coarse segmentation is predicted using an orthogonal network that processes the whole 3D volume slice by slice. In stage-II, the labels from stage-I are used to crop the region of interest (ROI) containing only the tumor region and seven orthogonal networks are used to predict a fine segmentation label for the given ROI. The final segmentation label is estimated using the averaged probability of all eight predictions (one in stage-I and seven in stage-II). A single residual Unet was used for stage-I and seven different orthogonal encoder-decoder networks were used for stage-II. Heavy data augmentation consisting of geometric transformation and random contrast was used to avoid overfitting and improve the generalization.

Team METU FL [48]

This team used an adaptation of previous work that decays the number of epochs in each FL round according to the relative difference between the initial loss and current round loss. Moreover, the learning rate was decayed by a fixed factor if there was no improvement in the mean DSC score for a patience number of rounds. Federated Averaging with server momentum was employed for the aggregation function with a momentum factor of 0.9 and an aggregator learning rate of 1. All collaborators participated in all FL rounds through the training.

Team MIP-FED [3]

The federated training algorithm combines variable local epochs, a decaying learning rate, and an ensemble weight aggregation function. The number of local training epochs performed in each federated round was varied, with high amounts of local

training epochs in the middle of federated training (up to 5 epochs per round) and low amounts of local training epochs in the beginning and end of federated training (0.5 epochs per round). The learning rate linearly decayed over the course of training, starting at 5e-3 in the first federated round and ending at 1e-6 in the last federated round. Finally, a weighted ensemble of three weight averaging methods was used: the weighted arithmetic mean (50%), median (10%), and geometric median (40%).

Team MIP-TRAVEL [49]

The general idea of the proposed traveling model is to train a single model sequentially, at one collaborator per time. The model is initialized at the first collaborator of the sequence. When training is completed, the model with the learned weights travels to the next collaborator. This process continues until the model finishes training at the last collaborator of the sequence, completing one cycle. A cycle consists of visiting every collaborator of the sequence once. In order to improve the model performance, the traveling process may be repeated for multiple cycles, which means that the model travels to each collaborator numerous times.

Team MiRL [4]

Their algorithm proposes a selection criterion for training collaborators in federated learning. The collaborators are sorted based on validation scores derived from local model training, combining the DSC score and Hausdorff distance in a linear score. After each round, the top-k collaborators are chosen based on this score for each tumor region to ensure the representation of all classes in training. Initially, all collaborators undergo training for the first few rounds to learn low-level features. Subsequently, top-performing collaborators are selected for aggregation rounds, with periodic training of all collaborators to prevent forgetting. The aggregation algorithm employed is weighted average aggregation. While all collaborators are chosen for aggregation in the initial rounds, only top performers are selected in consecutive rounds based on the defined validation score.

Supplementary Table 5: Participation and team details for the FeTS challenge 2021 (10 teams in total). The model ID is given in the Task 2 columns. Only a single affiliation is given for consecutive authors with the same institution.

**Team**	Task 1	Task 2	**Members**
Alpaca	Yes	A3	Sahil Nalawade*; Chandan Ganesh Bangalore Yogananda; Ben Wagner; Divya Reddy; Yudhajit Das; Fang Yu (University of Texas Southwestern Medical Center); Baowei Fei (University of Texas at Dallas); Ananth Madhuranthakam (Wake Forest School of Medicine); Joseph Maldjian (University of Texas Southwestern Medical Center)
BCN-AIM	Yes	-	Akis Linardos*; Kaisar Kushibar; Karim Lekadir (University of Barcelona)
CUHK	Yes	A1	Youtan Yin (Zhejiang University); Hongzheng Yang (Beihang University); Quande Liu*; Meirui Jiang; Cheng Chen; Qi Dou; Pheng-Ann Heng (The Chinese University of Hong Kong)
HT-TUAS	Yes	-	Muhammad Irfan Khan*; mojtaba jafaritadi; Esa Alhoniemi; Elina kontio (Turku University of Applied Sciences); Suleiman Khan (Amazon)
ibbm	Yes	-	Leon Mächler (ENS Paris)*; Ivan Ezhov; Suprosanna Shit; Johannes C. Paetzold; Florian Kofler; Timo Loehr; Benedikt Wiestler; Bjoern Menze (Techinical University of Munich)
mbi	-	A2	Kamlesh Pawar*; Shenjun Zhong; Zhaolin Chen; Gary Egan (Monash Biomedical Imaging, Monash University)
METU FL	Yes	-	Ece Isik Polat*; Gorkem Polat; Altan Kocyigit; Alptekin Temizel (Middle East Technical University)
MIP-FED	Yes	-	Anup Tuladhar (Department of Radiology & Hotchkiss Brain Institute, University of Calgary)*; Lakshay Tyagi (Indian Institute of Technology, Kanpur); Raissa Souza; Nils Daniel Forkert (Department of Radiology & Hotchkiss Brain Institute, University of Calgary)
MIP-TRAVEL	Yes	-	Raissa Souza*; Anup Tuladhar (Department of Radiology & Hotchkiss Brain Institute, University of Calgary); Pauline Mouches; Matthias Wilms (University of Calgary); Lakshay Tyagi (Indian Institute of Technology); Nils Daniel Forkert (Department of Radiology & Hotchkiss Brain Institute, University of Calgary)
MiRL	Yes	-	Vikas Kumar Anand*; Vishruth Shambhat (Indian Institute of Technology Madras); Akansh Maurya (Robert Bosch Centre for Data Science and AI); Ganapathy Krishnamurthi; Shubham Subhas Danannavar; Rohit Kalla (Indian Institute of Technology Madras)

References

[1] Pati, S., Baid, U., Edwards, B., Sheller, M., Wang, S.-H., Reina, G.A., Foley, P., Gruzdev, A., Karkada, D., Davatzikos, C., Sako, C., Ghodasara, S., Bilello, M., Mohan, S., Vollmuth, P., Brugnara, G., Preetha, C.J., Sahm, F., Maier-Hein, K., Zenk, M., Bendszus, M., Wick, W., Calabrese, E., Rudie, J., Villanueva-Meyer, J., Cha, S., Ingalhalikar, M., Jadhav, M., Pandey, U., Saini, J., Garrett, J., Larson, M., Jeraj, R., Currie, S., Frood, R., Fatania, K., Huang, R.Y., Chang, K., Quintero, C.B., Capellades, J., Puig, J., Trenkler, J., Pichler, J., Necker, G., Haunschmidt, A., Meckel, S., Shukla, G., Liem, S., Alexander, G.S., Lombardo, J., Palmer, J.D., Flanders, A.E., Dicker, A.P., Sair, H.I., Jones, C.K., Venkataraman, A., Jiang, M., So, T.Y., Chen, C., Heng, P.A., Dou, Q., Kozubek, M., Lux, F., Michálek, J., Matula, P., Keřkovský, M., Kopřivová, T., Dostál, M., Vybíhal, V., Vogelbaum, M.A., Mitchell, J.R., Farinhas, J., Maldjian, J.A., Yogananda, C.G.B., Pinho, M.C., Reddy, D., Holcomb, J., Wagner, B.C., Ellingson, B.M., Cloughesy, T.F., Raymond, C., Oughourlian, T., Hagiwara, A., Wang, C., To, M.-S., Bhardwaj, S., Chong, C., Agzarian, M., Falcão, A.X., Martins, S.B., Teixeira, B.C.A., Sprenger, F., Menotti, D., Lucio, D.R., LaMontagne, P., Marcus, D., Wiestler, B., Kofler, F., Ezhov, I., Metz, M., Jain, R., Lee, M., Lui, Y.W., McKinley, R., Slotboom, J., Radojewski, P., Meier, R., Wiest, R., Murcia, D., Fu, E., Haas, R., Thompson, J., Ormond, D.R., Badve, C., Sloan, A.E., Vadmal, V., Waite, K., Colen, R.R., Pei, L., Ak, M., Srinivasan, A., Bapuraj, J.R., Rao, A., Wang, N., Yoshiaki, O., Moritani, T., Turk, S., Lee, J., Prabhudesai, S., Morón, F., Mandel, J., Kamnitsas, K., Glocker, B., Dixon, L.V.M., Williams, M., Zampakis, P., Panagiotopoulos, V., Tsiganos, P., Alexiou, S., Haliassos, I., Zacharaki, E.I., Moustakas, K., Kalogeropoulou, C., Kardamakis, D.M., Choi, Y.S., Lee, S.-K., Chang, J.H., Ahn, S.S., Luo, B., Poisson, L., Wen, N., Tiwari, P., Verma, R., Bareja, R., Yadav, I., Chen, J., Kumar, N., Smits, M., Voort, S.R., Alafandi, A., Incekara, F., Wijnenga, M.M.J., Kapsas, G., Gahrmann, R., Schouten, J.W., Dubbink, H.J., Vincent, A.J.P.E., Bent, M.J., French, P.J., Klein, S., Yuan, Y., Sharma, S., Tseng, T.-C., Adabi, S., Niclou, S.P., Keunen, O., Hau, A.-C., Vallières, M., Fortin, D., Lepage, M., Landman, B., Ramadass, K., Xu, K., Chotai, S., Chambless, L.B., Mistry, A., Thompson, R.C., Gusev, Y., Bhuvaneshwar, K., Sayah, A., Bencheqroun, C., Belouali, A., Madhavan, S., Booth, T.C., Chelliah, A., Modat, M., Shuaib, H., Dragos, C., Abayazeed, A., Kolodziej, K., Hill, M., Abbassy, A., Gamal, S., Mekhaimar, M., Qayati, M., Reyes, M., Park, J.E., Yun, J., Kim, H.S., Mahajan, A., Muzi, M., Benson, S., Beets-Tan, R.G.H., Teuwen, J., Herrera-Trujillo, A., Trujillo, M., Escobar, W., Abello, A., Bernal, J., Gómez, J., Choi, J., Baek, S., Kim, Y., Ismael, H., Allen, B., Buatti, J.M., Kotrotsou, A., Li, H., Weiss, T., Weller, M., Bink, A., Pouymayou, B., Shaykh, H.F., Saltz, J., Prasanna, P., Shrestha, S., Mani, K.M., Payne, D., Kurc, T., Pelaez, E., Franco-Maldonado, H., Loayza, F., Quevedo, S., Guevara, P., Torche, E., Mendoza, C., Vera, F., Ríos, E., López, E., Velastin, S.A., Ogbole, G., Soneye, M., Oyekunle, D., Odafe-Oyibotha, O., Osobu, B., Shu'aibu, M., Dorcas, A., Dako, F., Simpson, A.L., Hamghalam, M., Peoples, J.J., Hu, R., Tran, A., Cutler, D., Moraes, F.Y., Boss, M.A., Gimpel, J., Veettil, D.K.,

Schmidt, K., Bialecki, B., Marella, S., Price, C., Cimino, L., Apgar, C., Shah, P., Menze, B., Barnholtz-Sloan, J.S., Martin, J., Bakas, S.: Federated learning enables big data for rare cancer boundary detection. Nature Communications **13**(1), 7346 (2022) https://doi.org/10.1038/s41467-022-33407-5 . Number: 1 Publisher: Nature Publishing Group. Accessed 2022-12-14

[2] Jiang, M., Yang, H., Zhang, X., Zhang, S., Dou, Q.: Efficient federated tumor segmentation via parameter distance weighted aggregation and client pruning. In: International MICCAI Brainlesion Workshop, pp. 161–172 (2022). Springer

[3] Tuladhar, A., Tyagi, L., Souza, R., Forkert, N.D.: Federated learning using variable local training for brain tumor segmentation. In: International MICCAI Brainlesion Workshop, pp. 392–404 (2021). Springer

[4] Shambhat, V., Maurya, A., Danannavar, S.S., Kalla, R., Anand, V.K., Krishnamurthi, G.: A study on criteria for training collaborator selection in federated learning. In: International MICCAI Brainlesion Workshop, pp. 470–480 (2021). Springer

[5] Wiesenfarth, M., Reinke, A., Landman, B.A., Eisenmann, M., Saiz, L.A., Cardoso, M.J., Maier-Hein, L., Kopp-Schneider, A.: Methods and open-source toolkit for analyzing and visualizing challenge results. Scientific Reports **11**(1), 1–15 (2021)

[6] Maier-Hein, L., Eisenmann, M., Reinke, A., Onogur, S., Stankovic, M., Scholz, P., Arbel, T., Bogunovic, H., Bradley, A.P., Carass, A., *et al.*: Why rankings of biomedical image analysis competitions should be interpreted with care. Nature communications **9**(1), 1–13 (2018)

[7] Maier-Hein, L., Menze, B., et al.: Metrics reloaded: Pitfalls and recommendations for image analysis validation. arXiv. org (2206.01653) (2022)

[8] Luu, H.M., Park, S.-H.: Extending nn-unet for brain tumor segmentation. In: International MICCAI Brainlesion Workshop, pp. 173–186 (2021). Springer

[9] Yuan, Y.: Evaluating scale attention network for automatic brain tumor segmentation with large multi-parametric mri database. In: International MICCAI Brainlesion Workshop, pp. 42–53 (2021). Springer

[10] Futrega, M., Milesi, A., Marcinkiewicz, M., Ribalta, P.: Optimized u-net for brain tumor segmentation. In: International MICCAI Brainlesion Workshop, pp. 15–29 (2021). Springer

[11] Ma, J., Chen, J.: Nnunet with region-based training and loss ensembles for brain tumor segmentation. In: International MICCAI Brainlesion Workshop, pp. 421–430 (2021). Springer

[12] Kotowski, K., Adamski, S., Machura, B., Zarudzki, L., Nalepa, J.: Coupling nnu-nets with expert knowledge for accurate brain tumor segmentation from mri. In: International MICCAI Brainlesion Workshop, pp. 197–209 (2021). Springer

[13] Jia, H., Bai, C., Cai, W., Huang, H., Xia, Y.: Hnf-netv2 for brain tumor segmentation using multi-modal mr imaging. In: International MICCAI Brainlesion Workshop, pp. 106–115 (2021). Springer

[14] Dobko, M., Kolinko, D.-I., Viniavskyi, O., Yelisieiev, Y.: Combining cnns with transformer for multimodal 3d mri brain tumor segmentation. In: International MICCAI Brainlesion Workshop, pp. 232–241 (2021). Springer

[15] Alam, S., Halandur, B., Mana, P.P., Goplen, D., Lundervold, A., Lundervold, A.S.: Brain tumor segmentation from multiparametric mri using a multi-encoder u-net architecture. In: International MICCAI Brainlesion Workshop, pp. 289–301 (2021). Springer

[16] Nguyen-Truong, H., Pham, Q.-D.: Dice focal loss with resnet-like encoder-decoder architecture in 3d brain tumor segmentation. In: International MICCAI Brainlesion Workshop, pp. 97–105 (2021). Springer

[17] Fidon, L., Shit, S., Ezhov, I., Paetzold, J.C., Ourselin, S., Vercauteren, T.: Generalized wasserstein dice loss, test-time augmentation, and transformers for the brats 2021 challenge. In: International MICCAI Brainlesion Workshop, pp. 187–196 (2021). Springer

[18] Yang, Y., Wei, S., Zhang, D., Yan, Q., Zhao, S., Han, J.: Hierarchical and global modality interaction for brain tumor segmentation. In: International MICCAI Brainlesion Workshop, pp. 441–450 (2021). Springer

[19] Jiang, Z., Zhao, C., Liu, X., Linguraru, M.G.: Brain tumor segmentation in multi-parametric magnetic resonance imaging using model ensembling and super-resolution. In: International MICCAI Brainlesion Workshop, pp. 125–137 (2021). Springer

[20] Wu, H.-Y., Lin, Y.-L.: Hardnet-bts: A harmonic shortcut network for brain tumor segmentation. In: International MICCAI Brainlesion Workshop, pp. 261–271 (2021). Springer

[21] Zeineldin, R.A., Karar, M.E., Mathis-Ullrich, F., Burgert, O.: Ensemble cnn networks for gbm tumors segmentation using multi-parametric mri. In: International MICCAI Brainlesion Workshop, pp. 473–483 (2021). Springer

[22] Carré, A., Deutsch, E., Robert, C.: Automatic brain tumor segmentation with a bridge-unet deeply supervised enhanced with downsampling pooling combination, atrous spatial pyramid pooling, squeeze-and-excitation and evonorm. In: International MICCAI Brainlesion Workshop, pp. 253–266 (2021). Springer

[23] Pnev, S., Groza, V., Tuchinov, B., Amelina, E., Pavlovskiy, E., Tolstokulakov, N., Amelin, M., Golushko, S., Letyagin, A.: Brain tumor segmentation with self-supervised enhance region post-processing. In: International MICCAI Brainlesion Workshop, pp. 267–275 (2021). Springer

[24] Feng, X., Bai, H., Kim, D., Maragkos, G., Machaj, J., Kellogg, R.: Brain tumor segmentation with patch-based 3d attention unet from multi-parametric mri. In: International MICCAI Brainlesion Workshop, pp. 90–96 (2021). Springer

[25] Singh, H.S.: Brain tumor segmentation using attention activated u-net with positive mining. In: International MICCAI Brainlesion Workshop, pp. 431–440 (2021). Springer

[26] Pawar, K., Zhong, S., Goonatillake, D.S., Egan, G., Chen, Z.: Orthogonal-nets: A large ensemble of 2d neural networks for 3d brain tumor segmentation. In: International MICCAI Brainlesion Workshop, pp. 54–67 (2021). Springer

[27] Bukhari, S.T., Mohy-ud-Din, H.: E1d3 u-net for brain tumor segmentation: submission to the rsna-asnr-miccai brats 2021 challenge. In: International MICCAI Brainlesion Workshop, pp. 276–288 (2021). Springer

[28] Milesi, A., Futrega, M., Marcinkiewicz, M., Ribalta, P.: Brain tumor segmentation using neural network topology search. In: International MICCAI Brainlesion Workshop, pp. 366–376 (2021). Springer

[29] Demoustier, M., Khemir, I., Nguyen, Q.D., Martin-Gaffé, L., Boutry, N.: Residual 3d u-net with localization for brain tumor segmentation. In: International MICCAI Brainlesion Workshop, pp. 389–399 (2021). Springer

[30] Shah, D., Biswas, A., Sonpatki, P., Chakravarty, S., Shah, N.: Neural network based brain tumor segmentation. In: International MICCAI Brainlesion Workshop, pp. 324–333 (2021). Springer

[31] Li, Z., Shen, Z., Wen, J., He, T., Pan, L.: Automatic brain tumor segmentation using multi-scale features and attention mechanism. In: International MICCAI Brainlesion Workshop, pp. 216–226 (2021). Springer

[32] Akbar, A.S., Fatichah, C., Suciati, N.: Unet3d with multiple atrous convolutions attention block for brain tumor segmentation. In: International MICCAI Brainlesion Workshop, pp. 182–193 (2021). Springer

[33] Yang, H., Shen, Z., Li, Z., Liu, J., Xiao, J.: Combining global information with topological prior for brain tumor segmentation. In: International MICCAI Brainlesion Workshop, pp. 204–215 (2021). Springer

[34] Maurya, S., Kumar Yadav, V., Agarwal, S., Singh, A.: Brain tumor segmentation in mpmri scans (brats-2021) using models based on u-net architecture. In:

International MICCAI Brainlesion Workshop, pp. 312–323 (2021). Springer

[35] Lin, W.-W., Li, T., Huang, T.-M., Lin, J.-W., Yueh, M.-H., Yau, S.-T.: A two-phase optimal mass transportation technique for 3d brain tumor detection and segmentation. In: International MICCAI Brainlesion Workshop, pp. 400–409 (2021). Springer

[36] Roth, J., Keller, J., Franke, S., Neumuth, T., Schneider, D.: Multi-plane unet++ ensemble for glioblastoma segmentation. In: International MICCAI Brainlesion Workshop, pp. 285–294 (2021). Springer

[37] Saueressig, C., Berkley, A., Munbodh, R., Singh, R.: A joint graph and image convolution network for automatic brain tumor segmentation. In: International MICCAI Brainlesion Workshop, pp. 356–365 (2021). Springer

[38] Yan, B.B., Wei, Y., Jagtap, J.M.M., Moassefi, M., Garcia, D.V.V., Singh, Y., Vahdati, S., Faghani, S., Erickson, B.J., Conte, G.M.: Mri brain tumor segmentation using deep encoder-decoder convolutional neural networks. In: International MICCAI Brainlesion Workshop, pp. 80–89 (2021). Springer

[39] Hsu, C., Chang, C., Chen, T.W., Tsai, H., Ma, S., Wang, W.: Brain tumor segmentation (brats) challenge short paper: Improving three-dimensional brain tumor segmentation using segresnet and hybrid boundary-dice loss. In: International MICCAI Brainlesion Workshop, pp. 334–344 (2021). Springer

[40] Druzhinina, P., Kondrateva, E., Bozhenko, A., Yarkin, V., Sharaev, M., Kurmukov, A.: Brats2021: Exploring each sequence in multi-modal input for baseline u-net performance. In: International MICCAI Brainlesion Workshop, pp. 194–203 (2021). Springer

[41] Karargyris, A., Umeton, R., Sheller, M.J., Aristizabal, A., George, J., Wuest, A., Pati, S., Kassem, H., Zenk, M., Baid, U., Narayana Moorthy, P., Chowdhury, A., Guo, J., Nalawade, S., Rosenthal, J., Kanter, D., Xenochristou, M., Beutel, D.J., Chung, V., Bergquist, T., Eddy, J., Abid, A., Tunstall, L., Sanseviero, O., Dimitriadis, D., Qian, Y., Xu, X., Liu, Y., Goh, R.S.M., Bala, S., Bittorf, V., Puchala, S.R., Ricciuti, B., Samineni, S., Sengupta, E., Chaudhari, A., Coleman, C., Desinghu, B., Diamos, G., Dutta, D., Feddema, D., Fursin, G., Huang, X., Kashyap, S., Lane, N., Mallick, I., Mascagni, P., Mehta, V., Moraes, C.F., Natarajan, V., Nikolov, N., Padoy, N., Pekhimenko, G., Reddi, V.J., Reina, G.A., Ribalta, P., Singh, A., Thiagarajan, J.J., Albrecht, J., Wolf, T., Miller, G., Fu, H., Shah, P., Xu, D., Yadav, P., Talby, D., Awad, M.M., Howard, J.P., Rosenthal, M., Marchionni, L., Loda, M., Johnson, J.M., Bakas, S., Mattson, P.: Federated benchmarking of medical artificial intelligence with MedPerf. Nature Machine Intelligence, 1–12 (2023) https://doi.org/10.1038/s42256-023-00652-2 . Publisher: Nature Publishing Group. Accessed 2023-07-18

[42] Nalawade, S., Ganesh, C., Wagner, B., Reddy, D., Das, Y., Yu, F.F., Fei, B.,

Madhuranthakam, A.J., Maldjian, J.A.: Federated learning for brain tumor segmentation using mri and transformers. In: International MICCAI Brainlesion Workshop, pp. 444–454 (2021). Springer

[43] Linardos, A., Kushibar, K., Lekadir, K.: Center dropout: A simple method for speed and fairness in federated learning. In: International MICCAI Brainlesion Workshop, pp. 481–493 (2021). Springer

[44] Yin, Y., Yang, H., Liu, Q., Jiang, M., Chen, C., Dou, Q., Heng, P.-A.: Efficient federated tumor segmentation via normalized tensor aggregation and client pruning. In: International MICCAI Brainlesion Workshop, pp. 433–443 (2021). Springer

[45] Khan, M.I., Jafaritadi, M., Alhoniemi, E., Kontio, E., Khan, S.A.: Adaptive weight aggregation in federated learning for brain tumor segmentation. In: International MICCAI Brainlesion Workshop, pp. 455–469 (2021). Springer

[46] Mächler, L., Ezhov, I., Kofler, F., Shit, S., Paetzold, J.C., Loehr, T., Zimmer, C., Wiestler, B., Menze, B.H.: Fedcostwavg: A new averaging for better federated learning. In: International MICCAI Brainlesion Workshop, pp. 383–391 (2021). Springer

[47] Pawar, K., Zhong, S., Chen, Z., Egan, G.: Brain tumor segmentation using two-stage convolutional neural network for federated evaluation. In: International MICCAI Brainlesion Workshop, pp. 494–505 (2021). Springer

[48] Isik-Polat, E., Polat, G., Kocyigit, A., Temizel, A.: Evaluation and analysis of different aggregation and hyperparameter selection methods for federated brain tumor segmentation. In: International MICCAI Brainlesion Workshop, pp. 405–419 (2021). Springer

[49] Souza, R., Tuladhar, A., Mouches, P., Wilms, M., Tyagi, L., Forkert, N.D.: Multi-institutional travelling model for tumor segmentation in mri datasets. In: International MICCAI Brainlesion Workshop, pp. 420–432 (2021). Springer
