# Supplementary material for: Towards fair decentralized benchmarking of healthcare AI algorithms with the Federated Tumor Segmentation (FeTS) challenge
Source: Nat Commun. 2025 Jul 8;16:6274. doi: 10.1038/s41467-025-60466-1 (PMC12238412; doi:10.1038/s41467-025-60466-1)
Supplement: Supplementary file 2 — Reporting Summary [file 41467_2025_60466_MOESM2_ESM.pdf]

Corresponding author(s): Spyridon Bakas

Last updated by author(s): Mar 28, 2025

Reporting Summary

Nature Portfolio wishes to improve the reproducibility of the work that we publish. This form provides structure for consistency and transparency in reporting. For further information on Nature Portfolio policies, see our Editorial Policies and the Editorial Policy Checklist.

Statistics

For all statistical analyses, confirm that the following items are present in the figure legend, table legend, main text, or Methods section.

n/a	Confirmed	
☐	☒	The exact sample size (*n*) for each experimental group/condition, given as a discrete number and unit of measurement
☐	☒	A statement on whether measurements were taken from distinct samples or whether the same sample was measured repeatedly
☒	☐	The statistical test(s) used AND whether they are one- or two-sided<br>*Only common tests should be described solely by name; describe more complex techniques in the Methods section.*
☒	☐	A description of all covariates tested
☒	☐	A description of any assumptions or corrections, such as tests of normality and adjustment for multiple comparisons
☐	☒	A full description of the statistical parameters including central tendency (e.g. means) or other basic estimates (e.g. regression coefficient) AND variation (e.g. standard deviation) or associated estimates of uncertainty (e.g. confidence intervals)
☒	☐	For null hypothesis testing, the test statistic (e.g. *F*, *t*, *r*) with confidence intervals, effect sizes, degrees of freedom and *P* value noted<br>*Give P values as exact values whenever suitable.*
☒	☐	For Bayesian analysis, information on the choice of priors and Markov chain Monte Carlo settings
☒	☐	For hierarchical and complex designs, identification of the appropriate level for tests and full reporting of outcomes
☒	☐	Estimates of effect sizes (e.g. Cohen's *d*, Pearson's *r*), indicating how they were calculated

Our web collection on statistics for biologists contains articles on many of the points above.

Software and code

Policy information about availability of computer code

Data collection	The data used in this project were collected at the radiology department of each collaborating institution, by directly accessing their local PACS. No additional data was used for data collection. The information of the exact PACS provider was not provided by each site.
Data analysis	Data analysis of the extracted scans was performed using the FeTS tool (v0.0.7). The source code for this package and its dependencies is available at https://github.com/FeTS-AI/Front-End<br>The local evaluation at each site was performed using MedPerf (branch fets-challenge, commit 9ff69e0): https://github.com/mlcommons/medperf/tree/fets-challenge/scripts<br>After collecting results data, python scripts using the pandas, seaborn and matplotlib libraries were used to analyse the challenge results and create illustrations and tables. The code used for the execution and analysis of the challenge is available on https://github.com/FETS-AI/Challenge

For manuscripts utilizing custom algorithms or software that are central to the research but not yet described in published literature, software must be made available to editors and reviewers. We strongly encourage code deposition in a community repository (e.g. GitHub). See the Nature Portfolio guidelines for submitting code & software for further information.

Data

Policy information about availability of data

All manuscripts must include a data availability statement. This statement should provide the following information, where applicable:

- Accession codes, unique identifiers, or web links for publicly available datasets
- A description of any restrictions on data availability
- For clinical datasets or third party data, please ensure that the statement adheres to our policy

The training and validation data of the FeTS challenge have been deposited in the Synapse platform under accession code \url{https://www.synapse.org/Synapse:syn54079892/wiki/626854} (registration required for download) and, as they are identical to the BraTS 2021 data, are also available via TCIA under accession code \url{https://www.cancerimagingarchive.net/analysis-result/rsna-asnr-miccai-brats-2021/} (free access). The reference segmentations for the validation data as well as the centralized testing data for the challenge are protected and are not available because they will be re-used in future competitions, which are only fair if evaluation sets are not public. Furthermore, decentralized testing data from the federated institutions are protected and are not available due to data sharing restrictions of the individual institutions.
The challenge results data generated in this study are published as a source data file. The source data file contains raw data underlying each figure, two example training cases, and the full challenge metric results for both tasks. Source data are provided with this paper.

Research involving human participants, their data, or biological material

Policy information about studies with human participants or human data. See also policy information about sex, gender (identity/presentation), and sexual orientation and race, ethnicity and racism.

Reporting on sex and gender	No sex- and gender-based analyses were performed, because the FeTS Challenge focused on algorithms delineating brain tumors irrespective of sex.
Reporting on race, ethnicity, or other socially relevant groupings	The FeTS Challenge included data from diverse populations from geographical locations on five continents, but no data on social groups was collected.
Population characteristics	All patients were diagnosed with brain tumors. We only have approximate population statistics for the Task 2 test set, which were reported by the federated data contributors. Patients age ranged from 5 to 94 years, with a mean of 56.9 years. The fraction of female patients was 39.7%.
Recruitment	Participants were recruited before the FeTS challenge study. Recruitment was different for each contributing institution.
Ethics oversight	Not applicable

Note that full information on the approval of the study protocol must also be provided in the manuscript.

Field-specific reporting

Please select the one below that is the best fit for your research. If you are not sure, read the appropriate sections before making your selection.

☒ Life sciences ☐ Behavioural & social sciences ☐ Ecological, evolutionary & environmental sciences

For a reference copy of the document with all sections, see nature.com/documents/nr-reporting-summary-flat.pdf

Life sciences study design

All studies must disclose on these points even when the disclosure is negative.

Sample size	The details of the number of patients in the challenge dataset can be in the methods section. 1251 cases were available for the challenge participants to develop their model. 219 cases were reserved for validation. The test datasets contained 570 cases for Task 1 (following the BraTS 2021 challenge split) and 2625 for Task 2. The number of samples for Task 2 was determined by availability of federated data contributors.
Data exclusions	The data required for this study required displaying the radiological features of glioblastoma scanned with multi-parametric MRI to characterize the anatomical tissue structure. Each case is specifically described by i) native T1-weighted (T1), ii) Gadolinium-enhanced T1-weighted (T1Gd), iii) T2-weighted (T2), and iv) T2-weighted-Fluid-Attenuated-Inversion-Recovery (T2-FLAIR) MRI scans. Cases with any of these sequences missing were not included in the study. Note that no inclusion/exclusion criterion applied relating to the type of acquisition (i.e., both 2D axial and 3D acquisitions were included, with a preference for 3D if available), or the exact type of sequence (e.g., MP-RAGE vs SPGR). The only exclusion criterion was for T1-FLAIR scans that were intentionally excluded to avoid mixing varying tissue appearance due to the type of sequence, across native T1-weighted scans. For the challenge Task 2, some test samples were excluded based on manual annotation quality control, as described in the results section.
Replication	The source code or containerized applications for challenge submissions can be re-run by the organizers at any time. Datasets are fixed and

Replication	the evaluation pipeline deterministic. Availability of the datasets used for Task 2 is, however, beyond the control of the organizers, as the federated institutions may manage the local MedPerf folders as they wish.
Randomization	For the data from the BraTS challenge, cases were split randomly into training, validation and test sets. Data collection in the federation was performed independently of the FeTS Challenge, so we had no influence on it.
Blinding	Blinding was not relevant for our study. The imaging data was collected independently by the federated institutions and the BraTS challenge organizers before this study. They were not allocated to groups during data collection or analysis. All algorithm submissions to the challenge were evaluated automatically using the same pipeline, so there were no groups here, either.

Reporting for specific materials, systems and methods

We require information from authors about some types of materials, experimental systems and methods used in many studies. Here, indicate whether each material, system or method listed is relevant to your study. If you are not sure if a list item applies to your research, read the appropriate section before selecting a response.

Materials & experimental systems

n/a	Involved in the study	
☒	☐	Antibodies
☒	☐	Eukaryotic cell lines
☒	☐	Palaeontology and archaeology
☒	☐	Animals and other organisms
☒	☐	Clinical data
☒	☐	Dual use research of concern
☒	☐	Plants

Methods

n/a	Involved in the study	
☒	☐	ChIP-seq
☒	☐	Flow cytometry
☐	☒	MRI-based neuroimaging

Plants

Seed stocks	n/a
Novel plant genotypes	n/a
Authentication	n/a

Magnetic resonance imaging

Experimental design

Design type	resting state
Design specifications	varies between contributing institutions. The FeTS Challenge used a dataset that was acquired by independent institutions before the challenge took place.
Behavioral performance measures	n/a

Acquisition

Imaging type(s)	structural
Field strength	depends on scanner, 1.5T or 3T
Sequence & imaging parameters	Each case in the FeTS Challenge contains four MRI sequences: i) native T1-weighted (T1), ii) Gadolinium-enhanced T1-weighted (T1Gd), iii) T2-weighted (T2), and iv) T2-weighted-Fluid-Attenuated-Inversion-Recovery (T2-FLAIR)
Area of acquisition	whole brain

Diffusion MRI ☐ Used ☒ Not used

Preprocessing

Preprocessing software	We performed DICOM to NIfTI conversion, co-registration to anatomical template, and brain extraction as described in the methods section. All the tools used for this study are open-sourced in https://github.com/FETS-AI/Front-End.
Normalization	n/a
Normalization template	n/a
Noise and artifact removal	n/a
Volume censoring	n/a

Statistical modeling & inference

Model type and settings	n/a
Effect(s) tested	n/a

Specify type of analysis: ☐ Whole brain ☐ ROI-based ☐ Both

Statistic type for inference (See Eklund et al. 2016)	n/a
Correction	n/a

Models & analysis

n/a	Involved in the study
☒	☐ Functional and/or effective connectivity
☒	☐ Graph analysis
☒	☐ Multivariate modeling or predictive analysis
